# Optimal Geometry for Focused Ion Beam-Milled Samples for Direct-Pull Micro-Tensile Testing Performed In Situ in a Scanning Electron Microscope

**DOI:** 10.3390/ma17215144

**Published:** 2024-10-22

**Authors:** Daniel B. Yin, Haiping Sun, Amit Misra

**Affiliations:** 1 Department of Materials Science and Engineering, University of Michigan, Ann Arbor, MI 48109, USA; 2 Sigma Division, Los Alamos National Laboratory, Los Alamos, NM 87545, USA; 3 Michigan Center for Materials Characterization, University of Michigan, Ann Arbor, MI 48109, USA; haipings@umich.edu; 4 Department of Mechanical Engineering, University of Michigan, Ann Arbor, MI 48109, USA

**Keywords:** micro-tensile testing, additive manufacturing, finite element analysis, FEA, dogbone geometry, focused ion beam milling, FIB, PI 89 PicoIndenter, copper–iron alloys

## Abstract

A thorough procedure was developed to efficiently manufacture dogbone samples using focused ion beam (FIB) milling for micro-tensile testing. A Bruker PI 89 PicoIndenter, Billerica, MA, USA, was used as a case study, although the analysis and results are applicable to other micro-mechanical testing systems capable of mounting a standard, Ø12.7 mm × Ø3.2 mm pin, scanning electron microscopy (SEM) pin stub (Ted Pella, Redding, CA, USA). Nine dogbones were made from an Fe-45Cu alloy additively manufactured using powder-fed laser-directed energy deposition (DED-LB). Testing showed that fracture was confined to the gauge section for all dogbones and that the fracture mode, ductile vs. brittle, was entirely dependent on the grain orientation relative to the loading direction. The analysis showed that the measured plastic strain to failure can vary from >11% (optimal geometry) to <1% (non-optimal geometry) in micro-tensile testing of high-tensile-strength (>1 GPa) metallic materials. Subsequently, a finite element analysis (FEA) was conducted to identify the improved dogbone geometries. A total of ten thousand dogbone geometries were tested, and their dimensions were defined by a set of four adjustable parameters (corner radius, load surface angle, load surface length, and dogbone head length). The gauge width and gauge length were fixed to 4 µm and 10 µm, respectively. Three-dimensional surface plots of the stress concentration as a function of two parameters were used to identify the optimal ranges of parameter values. The addition of maximum width and length constraints, measuring 25 µm and 30 µm, respectively, allowed us to identify an optimal geometry at load surface angles of 30° and 45°. Their respective dimensions (corner radius, load surface length, and dogbone head length) are, in µm, 12, 6, and 7 and 10, 7, and 7. Testing these two optimal geometries with a range of gauge lengths from 4 to 20 µm showed that smaller gauge lengths only slightly reduced the detrimental stress concentration outside the gauge section. However, smaller gauge lengths will notably improve the FIB surface polishing step as tapering is reduced with smaller dogbone lengths.

## 1. Introduction

Direct-pull micro-tensile (DPMT) testing is a natural evolution of micro-mechanical testing originally utilized for indentation and micro-pillar compression [1]. The precise displacement and/or load control of the transducer is merely operated in the reverse direction and FIB milling converts an indentation/compression tip into a tensile gripper.

Such a method can be applied to single crystalline materials as well as nanocrystalline alloys/composites to study the size effect on the plasticity [2,3,4,5]. And if the grains are large enough, micro-tensile testing can target grains with specific orientations/Schmid factors [6]. It has also been implemented on thin films where the substrate can be easily etched away [7,8]. Recently, this method has been applied towards selective laser remelted Al-Si alloys [9,10], a surface modification technique that often utilizes a laser additive manufacturing (AM) setup with the feedstock supply disabled. And as with this work, the volume of material available is a major constraint in obtaining tensile data, and DPMT lies perfectly between macro-scale and nano-scale transmission electron microcopy (TEM) push-to-pull tensile testing. The latter requires samples so small and thin that it generally measures the properties of just a single grain.

The material used in this work is a Fe-45Cu alloy made by laser powder-fed directed energy deposition (DED-LB) as described by Chatterjee et al. [11]. The slight deviation in composition from the starting equimolar powder mixture is due to the preferential evaporation of copper [12]. This AM technique flows the powder through a nozzle, coaxial to the laser beam, using a shielding gas. The original deposited sample was a roughly 1 cm^3^ cube. However, after sectioning with a diamond saw, the sample piece (~9 × 7 × 3 mm) is far too small to cut multiple tensile dogbone specimens from using computer numerical control (CNC) for macro-scale tensile testing. Nevertheless, micro-tensile testing serves as a direct comparison to the micro-pillar compressive testing previously performed by Chatterjee et al. [11] on the same sample. Similar compression tests were carried out on larger samples of a similar alloy composition by Zafari et al. [13] using a pulse laser powder bed fusion (PBF) setup.

**Figure 1:**
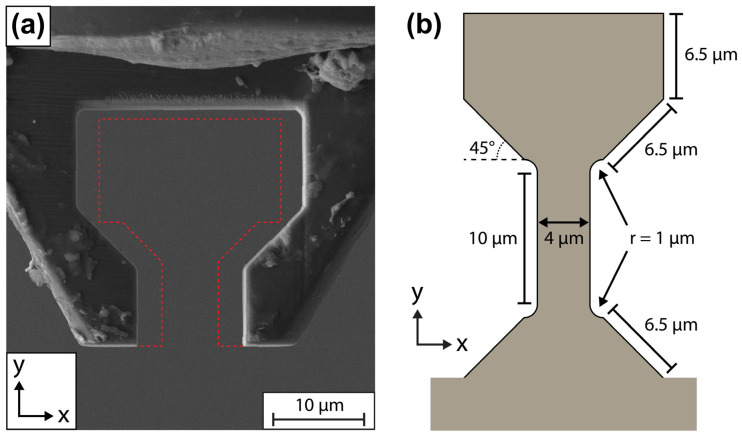
(**a**) SEM image of our FIB-milled diamond gripper. The red dotted lines highlight the additional material that was FIB-milled away to convert the gripper from the original geometry to the 45° optimal dogbone geometry identified at the end of this work. (**b**) A schematic of the original dogbone shape to be cut from our rectangular sample piece and its dimensions. The corner arcs have an arc angle of 90° and eliminate the normally sharp interface between the straight gauge and angled load surfaces.

This work will first explain step by step the complex procedure of fabricating the dogbone samples using FIB milling. At several points, the importance of the FIB patterning sequence and sample alignment and their connection to achieving good-quality dogbone samples and tensile tests will be brought up. Nine dogbone samples were made according to the geometry specified in Figure 1b. These dogbones were tested on a Bruker PI 89 PicoIndenter using a gripper manufactured by FIB milling from a diamond conical indenter tip (Figure 1a). The results of our tensile tests helped validate the soundness of our dogbone fabrication procedure.

Finally, a finite element analysis (FEA) was conducted to identify optimal dogbone geometries for two different load surface angles. While a previous study by Casari et al. [14] also utilized FEA to identify an optimal geometry for DPMT testing, they elected to fix their gauge width to 1.75 µm. This was an acceptable value for their single-phase, single-crystal Si and GaAs samples. However, for our multi-phase material, a small gauge width is not ideal. Scaling up their geometry to match our desired gauge width of 4 µm resulted in an overall dogbone length of over 70 µm that was impractical to make with FIB milling due to the difficulty in eliminating the taper along the loading direction. Therefore, new optimal geometries had to be identified accommodating a wider gauge width while maintaining a reasonable overall dogbone size.

## 2. Methodology—Micro-Tensile Dogbone Preparation

An Allied TechCut 4 low-speed saw with a diamond metal-bonded blade (Allied High Tech Products, Cerritos, CA, USA) was used to cut a roughly 9 × 7 × 3 mm rectangular piece from the DED-LB-printed 1 cm^3^ cube sample. This piece was then adhered to a 12.7 mm diameter, 8 mm pin height standard SEM pin stub using cyanoacrylate adhesive (superglue). The adhesive was applied uniformly along the four contact edges between the sample and the pin stub surface to ensure a strong bond. Afterwards, hand polishing was carried out in three steps: 320 grit silicon carbide paper, 9 µm diamond solution on a Struers MD Largo pad, and 3 µm diamond solution on a Struers MD Mol pad (Struers, Champigny-sur-Marne, France). Post-polishing, the sample was cleaned with water and isopropyl alcohol. Finally, silver paint was applied across the same four glued edges between the sample and the pin stub surface to eliminate charging of the sample during SEM.

Two dual-beam SEM/FIB microscopes (Thermo Fisher Scientific, Waltham, MA, USA) were used to prepare the dogbone samples, a Thermal Fisher Helios G4 Plasma FIB UXe (PFIB) and a Thermal Fisher Helios 650 Nanolab FIB (Ga+ FIB). The alignment marker, rough planing cuts, undercuts, and rectangular blank cuts were conducted exclusively on the PFIB as its microamp FIB current modes reduce the patterning time significantly.

### 2.1. Alignment Marker Cuts

**Figure 2:**
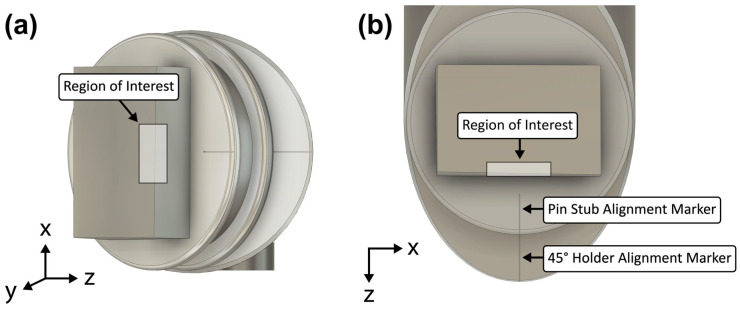
Schematic of the rectangular sample adhered to a standard pin stub mounted on a 45° holder. The two separately cut alignment markers on the pin stub and holder are visible in (**a**), looking straight downwards when the sample is mounted on the SEM/FIB stage. In (**b**), they appear coincident when the field of view is shifted to be perpendicular with the top surface of the sample (stage tilted +45° for SEM view or +7° for FIB view).

**Figure 3:**
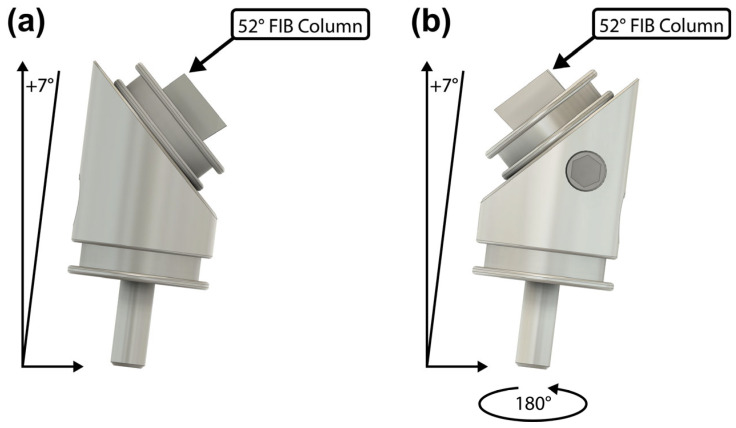
Schematic of the two sample orientations in the SEM/FIB when the pin stub is mounted on a 45° holder. Once the sample is aligned in one orientation, only 180° stage rotations are needed to switch back and forth. Orientation (**a**) is used for alignment marker cuts, planing cut 1, undercut 1, undercut 2, and the steps in Figure 5c. Orientation (**b**) is used for planing cut 2, rectangular blank cuts, and the steps in Figure 5a,b.

First, the PFIB (30 kV, 2.5 µA) was used to cut a long rectangular 50 × 2000 × 10 µm (length × width × depth) alignment marker on both the pin stub and the 45° holder (Figure 2). The stage was tilted to +7° and rotated until the pin stub surface was perpendicular to the FIB column (Figure 3a). This patterning was performed in 50 × 450 µm steps due to the field-of-view constraint of the PFIB. Each step has a small amount of overlap, ~50 µm, with the previous to ensure the overall rectangle is straight. The long length of the alignment marker assists with ensuring consistent rotational alignment about the y-axis when mounting the pin stub on both the Bruker PI 89 PicoIndenter’s pin stub holder (Bruker, Eden Prairie, MN, USA) before tensile experiments and the 45° holder afterwards. Figure 2b shows the two alignment markers and their coincidence when looking downwards perpendicular to the pin stub surface. For all figures, the axis labels’ directions match that of the PI 89’s control software (TriboScan ver. PI 10.1.0.43).

### 2.2. Rough Planing Cuts

**Figure 4:**
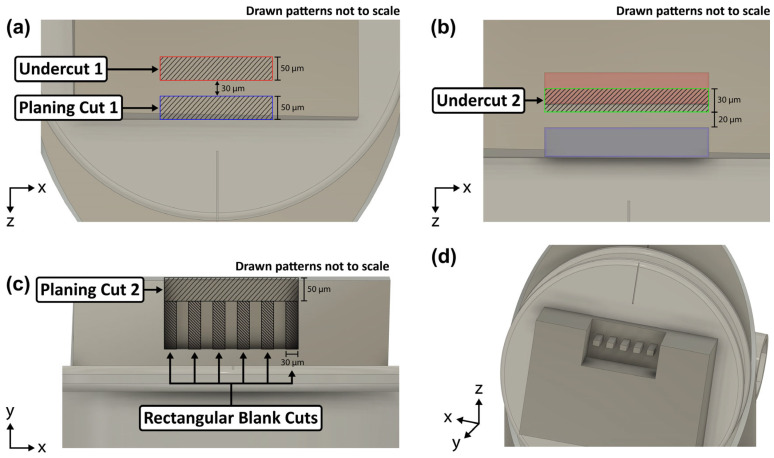
Schematic of the multi-step rough FIB patterning carried out exclusively on the PFIB. The cuts are drawn larger than actual for visual purposes. The red and blue filled rectangles in (**b**) represent the area cut away in (**a**). The rectangular blank cuts in (**c**) are spaced 30 µm apart. (**d**) The finished rectangular blanks orthogonal and aligned with the pin stub’s alignment marker.

The planing and undercuts were all conducted at 30 kV/2.5 µA. Planing cut 1 (Figure 4a) of roughly 50 × 800 × 100 µm (length × width × depth) was placed along the outside exposed edge of the sample. The 50 um length direction must be parallel to the alignment marker on the pin stub to prevent rotational misalignment about the y-axis (confer Figure 7c). Planing cuts are necessary for two reasons. Firstly, they ensure that the free-floating slab, from which the rectangular blanks are cut, will be coincident to the gripper when the pin stub is mounted on the PI 89 stage. Here, coincidence means no angular misalignment about the x-axis of rotation (confer Figure 7d). The hand-polishing step guarantees the top surface (9 × 7 mm) of the sample is near flat but not necessarily parallel with the pin stub surface. Second, the planing cuts also ensure the dogbone samples are fabricated away from any leftover surface damage caused by the cutting and polishing steps. This is especially important on the thin side surfaces (3 mm), which were cut by the diamond saw but could not be hand-polished.

Concurrent with planing cut 1, we also make undercut 1 (Figure 4a) with dimensions 50 × 800 × 100 µm (length × width × depth) set 30 µm away from the exposed surface of the corner ledge to create the free-floating slab. Then, undercut 2 (Figure 4b) with dimensions 30 × 800 × 100 µm (length × width × depth) is made to thin the slab down to 20 µm thick. This second undercut also ensures that the underside of the free-floating slab is actually cut to the original desired depth of 100 µm. The first undercut generally will not reach the desired depth as interior patterns have difficulty removing material compared to those with more than one surface exposed to vacuum.

Afterwards, the stage is rotated 180° so that the pin stub surface is parallel to the FIB column (Figure 3b). Here, planing cut 2 (Figure 4c) is made matching the dimensions of planing cut 1 except with a reduced depth of 30 µm. Once complete, the free-floating slab is recessed roughly 50 µm from both the original free surfaces of the sample.

The rectangular blanks are then cut from the free-floating slab at 30 kV/1 µA. These rectangular FIB patterns (Figure 4c) of 50 × 30 × 20 µm (length × width × depth) are spaced 30 µm apart to provide clearance for the gripper. It is important to first perform undercut 2 as a thinner slab causes less redeposition to occur along the sides of the rectangular blanks. Nevertheless, some redeposition still occurred and was removed with pairs of cross-section cleaning cuts placed along the two side edges of the rectangular blanks.

This concludes the rough FIB cuts (Figure 4d), and the subsequent FIB patterning to complete the dogbone samples was carried out using either the PFIB or the Ga+ FIB.

### 2.3. Sample Alignment Check

**Figure 5:**
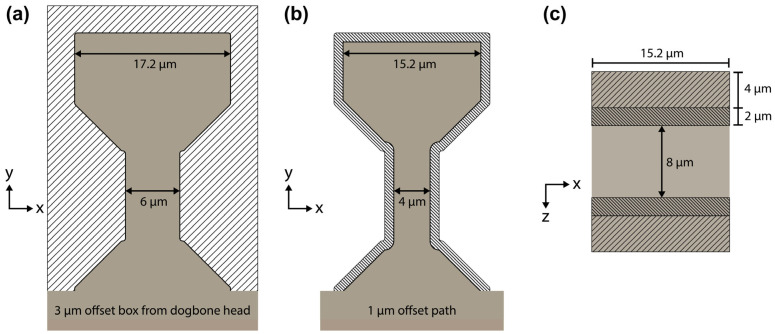
Schematic of the dogbone (**a**) shape cut, (**b**) edge polishing, and (**c**) thickness reduction (4 µm) + surface polishing (2 µm).

**Figure 6:**
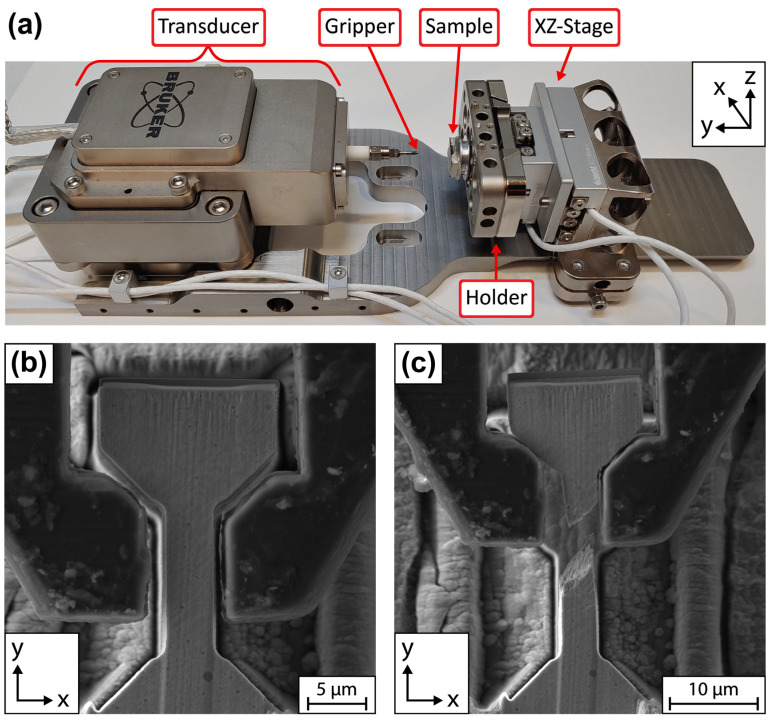
(**a**) The Bruker PI 89 PicoIndenter setup for micro-tensile testing. The FIB-milled diamond gripper was screwed onto the high-load transducer on the left. (**b**) An SEM image of a dogbone sample (dogbone 15) aligned inside the gripper and ready for tensile testing. (**c**) A corresponding image of the dogbone sample post-fracture. This fracture was relatively brittle and the ~45° fracture angle led to some movement of the dogbone head in the gripper after failure.

**Figure 7:**
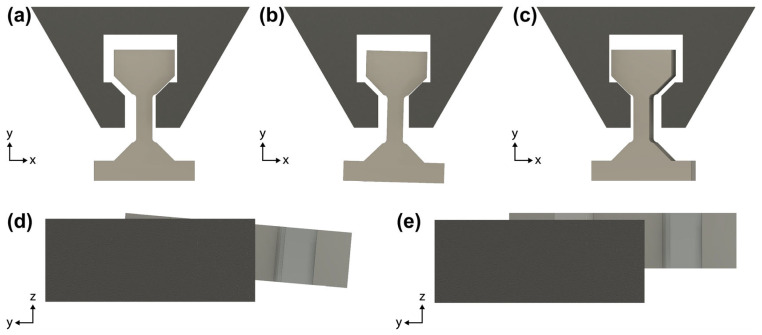
The five possible misalignments observed during tensile testing: (**a**) translational in x-axis, (**b**) rotational about z-axis, (**c**) rotational about y-axis, (**d**) rotational about x-axis, and (**e**) translational in z-axis. All misalignments shown are exaggerated for visual clarity.

Before proceeding to the shape and polishing patterns (Figure 5), the sample (on the pin stub without 45° holder) was mounted on the PI 89 (Figure 6a) and viewed in a Tescan MIRA3 FEG-SEM (Tescan Group, Brno, Czech Republic) to check for any rotational misalignment about the z-axis (Figure 7b). For the nine dogbones tested, the misalignment was 2.7° clockwise.

The source of this misalignment is the alignment marker cut into the 45° holder. The vertical centerline of the 45° holder can only be approximated using the circular measurement tool set to a diameter that closely matches the curvature of its outside edge when viewed in the SEM. This is because the SEM/FIBs used lack the field of view to encompass the entire holder. Thus, any angular error in the 45° holder’s alignment marker will propagate. The pin stub alignment marker was cut using the holder’s alignment marker as a guideline, which was then used to perform the alignment shown in Figure 3a.

When using a 45° holder, the measured misalignment must be corrected by rotating the FIB patterns in Figure 5a,b rather than the microscope stage. This is because the 45° holder tilts the sample out of the rotation plane of the microscope. Thus, a stage rotation that horizontally aligns the top flat edge of the rectangular blanks will produce some out-of-plane tilt (Figure 7c). This effect was observed in the SEM secondary electron (SE) mode as either the right or left side surface of the rectangular blanks will become brighter. If there was no misalignment, those side surfaces should not be visible except for a minor amount of taper.

A dogbone prepared without the use of a 45° holder does not require FIB pattern rotation to correct misalignment as the sample lies on the rotation plane of the stage. On the other hand, since the pin stub is now on the rotation plane of the stage, this type of misalignment is unlikely to occur in the first place. This is because the pin stub’s alignment marker now independently defines the vertical centerline that the dogbones will be orthogonal to.

Manufacturing dogbones without a 45° holder presents its own challenges as only the PFIB stage can tilt to −38° (parallel to the FIB column) to perform top-down dogbone shape pattern cuts (Figure 5a,b). The Ga+ FIB is limited to only a −10° tilt. Both FIBs, however, can perform the dogbone surface polishing steps (Figure 5c) at a +52° tilt, perpendicular to the FIB column.

### 2.4. Dogbone Shape Cut and Surface Polishing

Bitmap patterns designed in Adobe Illustrator were used in lieu of numerous rectangular cross-section cleaning and circular FIB patterns. This significantly reduced the complexity of the final patterning steps (Figure 5a,b). It also avoided the issue where the circular FIB patterns tended to overcut past their displayed outline more than their rectangular counterparts, requiring a difficult-to-judge offset placement. Finally, bitmap patterns ensure perfect symmetry compared to manually placed patterns.

Figure 5 shows the multi-step process for finishing the dogbone samples. Prior to starting the rough dogbone shape cut, the rectangular blank’s width is reduced from 30 µm to approximately 20 µm using a pair of rectangular cross-section cleaning patterns at 30 kV/15 nA in the PFIB or 30 kV/9 nA in the Ga+ FIB. Then, the rough dogbone shape (Figure 5a) was cut from the rectangular blank using the same voltage and current settings. The bitmap pattern for this rough cut was 23.2 µm wide and was created using an offset rectangle set 3 µm from the top and side edges of an enlarged dogbone head. The enlarged dogbone shape in Figure 5a was made to match the dogbone shape created by the 1 µm offset cutting path (Figure 5b) in the next dogbone patterning step. This enlarged dogbone shape compensated for the minor amount of overcutting that occurred at higher currents. Once the rough dogbone shape cut was verified as complete in the SEM view, the second dogbone edge polishing pattern (Figure 5b) was used to mill away the taper along the side edges of the dogbone. This second shape pattern was created at 30 kV/4 nA in the PFIB or 30 kV/2.1 nA in the Ga+ FIB and the cutting area was confined to a 1 µm wide strip along the side edges.

The taper reduction process can also be observed in the SEM as the second dogbone shape pattern will slowly cut an “outline” groove of the dogbone shape on the floor created by the undercuts in Figure 5a,b. Once the width of the gauge section of that “outline” reaches the desired gauge width, this step was complete.

After the shape cuts and edge polishing were completed, the stage was rotated 180° and rectangle FIB patterns were used (Figure 5c), first to reduce the dogbone thickness to approximately 12 µm, and then to polish the top and bottom surfaces. The targeted final dogbone thickness was around 8 µm. Thickness reduction was carried out at 30 kV/4 nA in the PFIB or 30 kV/2.1 nA in the Ga+ FIB while polishing was conducted at 30 kV/1 nA or 30 kV/0.79 nA, respectively.

Difficulties in efficiently achieving a taper-free surface along the gauge section come from the higher depth of material the beam must mill through. The areas of the dogbone head on both sides of the gauge section are easily polished flat by patterns with a depth set close to the overall length of the dogbone. In our experiments, this depth was approximately 21 µm. Meanwhile, the bottom part of the gauge remains thicker than its top half. To polish away this taper, two patterning strategies were employed using cross-section cleaning patterns.

(1) The first involved reducing the width of the FIB pattern from 15.2 µm to 4 µm, encompassing only the gauge section. This reduced width pattern was repeatedly ran until SEM snapshots showed the gauge section was taper-free on the bottom half. However, this method tended to form channels in the curved edges beside the gauge section on the bottom half of the dogbone. Subsequent full-width (15.2 µm) patterns can reduce the relative depth of these channels but would eventually recreate a tapered gauge section as the areas beside the gauge section mill faster.
(2) The second involved running a sequence of full-dogbone-width (15.2 µm) patterns with gradually decreasing cross-sectional height and increasing depth. The patterning started with a cross-sectional height of 1 µm and a depth of 40 µm (slightly greater than the overall dogbone length). Subsequent patterns had reduced cross-sectional heights of 0.5 µm and 0.25 µm paired with depths of 60 µm and 80 µm, respectively. In effect, the cross-sectional height was halved each step while the depth was increased in 50% increments over the original (1.5× and 2× dogbone length).

An alternative strategy using regular rectangle patterns was considered but ultimately not used for our experiments. Compared to cross-section cleaning patterns, it was found that regular rectangle patterns were less efficient at removing material. However, a rectangle pattern does not necessarily need to be rerun if SEM snapshots show the taper is still present. The pattern’s depth can simply be increased in situ to keep it running. Thus, using regular rectangle patterns could produce an equally good surface polish as compared to the two cross-section cleaning pattern strategies while requiring much less user intervention. Nevertheless, if this strategy is employed by other authors, it is still recommended to reduce the rectangle pattern’s cross-sectional height occasionally as the taper is reduced. This reduces the area of the pattern not actively milling the remaining taper and improves efficiency.

## 3. Methodology—Micro-Tensile Experiment

The pin stub containing the finished dogbones was mounted onto the Bruker PI 89’s detachable holder with the assistance of an optical microscope and a small parallel block. The optical microscope allowed a visual of the alignment marker that was rotated until it coincided with the edge of the parallel block placed against the side of the holder. This procedure is analogous to aligning the pin stub’s alignment marker on the 45° holder (Figure 2b), substituting the alignment marker on the 45° holder with the edge of the parallel block. The pin stub was then fixed with a set screw. While this loading method required a high degree of hand–eye coordination, the multiple sample loadings carried out in this work generally resulted in less than 2° of rotational misalignment (Figure 7c). Larger misalignments are easily spotted in the SEM and were corrected with another iteration of the alignment process just described.

For the PI 89, it is recommended that pin stubs with reduced pin lengths are used. This experiment was carried out using a standard 12.7 mm diameter, 8 mm pin length stub which had a pin length roughly 1.5 mm too tall for the PI 89’s detachable stage. This additional height results in the holder not seating onto the XZ-stage as the bottom of the pin protrudes. Raising the pin stub by 1.5 mm results in the pin stub no longer resting level against the surface of the holder. This increases the tendency for the pin stub to tilt when the set screw is tightened, resulting in large misalignments (Figure 7b,d) between the dogbone and the gripper that cannot be corrected using the stage controls. To rectify this issue, a pair of diagonal cutters was used to trim the excess length of the standard pin and then the cut surfaces were buffed to remove burs.

Once the pin stub is aligned in the optical microscope, the sample holder is slid into place onto the dovetail grooves of the XZ-stage and held in place by another set screw. This set screw is on the opposite side of the holder, as viewed from the perspective shown in Figure 6a. Another set screw for the XZ-stage, shown in the bottom right of Figure 6a, is loosened to slide the entire stage assembly closer to the gripper (~1.5 cm standoff) as the transducer has limited travel.

All the tensile tests were performed with the same displacement-controlled profile. The gripper displacement rate was set to 150 nm/s with a total displacement endpoint of 10 µm. Once the gripper had reached the set endpoint of 10 µm, the stage was lowered by 25 µm. This ensured the fracture surfaces were not destroyed. Finally, the gripper was returned to the starting position at 500 nm/s.

For the dogbone geometry, the displacement-rate-to-gauge-length ratio resulted in an initial strain rate of ~10^−2^. This is higher than in other works [3,5,6,7], which report values that generally range from 10^−3^ to 10^−4^, and is due to the reduced precision of the PI 89’s drift control in the tensile mode. Empirical observations during our tensile testing showed that the PI 89’s stage tends to drift transversely in the x-axis at a rate of roughly 100 nm/min. Thus, a higher displacement rate was necessary to ensure that the pull-to-fracture period was completed before sample drift led to notable non-uniaxial loading conditions caused by increasing misalignment. A recent manufacturer technical service (Bruker Technical Support Bulletin No. 2: Preventing Cable Damage for PI 89 Stages) removed the stiff transparent tubing installed over the two white cables controlling the XZ-stage and empirically eliminated the previously observed drift. All tensile tests in this work were conducted pre-service.

Video capture of the SEM view was used to identify the moment of fracture initiation as well as to relatively assess the degree of compliance of the system.

### 3.1. Potential Misalignments and Corrections

There are five types of misalignments that can exist between the dogbone and gripper. These misalignments, if not mitigated, can negatively affect the measured mechanical properties as loading is no longer perfectly uniaxial. Casari et al showed that even small misalignments can yield large errors in the Young’s modulus and strain [14]. The two translational misalignments, shown in Figure 7a,e, are corrected using the software stage controls. The z-axis rotational misalignment, shown in Figure 7b, is prevented by planing cut 2 if dogbone preparation is carried out without a 45° holder. Otherwise, it is likely some misalignment of this type will persist even after planing cut 2, which must be corrected using the method described in Section 2.3. The misalignment about the y-axis, shown in Figure 7c, is minimized by using the pin stub alignment marker when mounting the pin stub onto the PI 89 holder. The x-axis rotational misalignment, shown in Figure 7d, is prevented by planing cut 1, undercut 1, and undercut 2.

### 3.2. Post-Experiment—Broken Dogbone Head Extraction

Two methods for removing broken dogbone heads from the gripper were tested successfully.

(1) A vertical pillar was cut from the bulk material using the PFIB. Post-tension test, the gripper with the broken head was positioned directly above the pillar and lowered in small increments until the head was pushed out. Afterwards, the gripper was slowly retracted, and the pillar was passed through the central channel in the gripper. An alignment rectangle was also cut alongside the pillar as the broken dogbone head obscures the view of the vertical pillar below. Generally, once pushed out, electrostatic forces pulled the broken head onto the top surface of the gripper along the outside edges. This did not affect the measured force of future tension tests but was present in the recorded videos for those tests.
(2) The tip of a cotton swab was pinched using a pair of gloved fingers and twisted a couple of times to create a thin braid of cotton fibers. This braid was then wetted with acetone until drops were visible on the surface. Then, the braid was gently brushed along the top edge of the gripper containing the broken dogbone head. The adhesive force of the acetone having had its surface tension broken from contact with the gripper then dislodged the broken dogbone head. The procedure was carried out with the assistance of a high-magnification optical microscope that could resolve the gripper.

The second method was far more time-efficient, safe, and could be completed in less than a minute once the PI 89 had been removed from the SEM chamber. The first method required several hours of PFIB milling as a significant amount of material needed to be excavated. Additionally, the four sides of the pillar needed to be polished to reduce the taper that normally causes the pillar to widen with increasing FIB cut depth. This ensured that the pillar can still pass through the central channel of the gripper without becoming stuck after lifting the broken dogbone head out of the gripper. However, the first method can be carried out in situ, and will be very useful for high-temperature aging experiments with multiple dogbones where the chamber cannot be vented. The authors recommend the second method for all room-temperature experiments.

## 4. Results—Micro-Tensile Experiment

In total, nine dogbone samples were tested. The dogbone thickness ranged from roughly 4 μm to 10 μm; most were around 7.5 ± 1 μm. The dogbones with lower thicknesses were a product of difficulties in FIB surface polishing discussed in Section 2.4.

**Figure 8:**
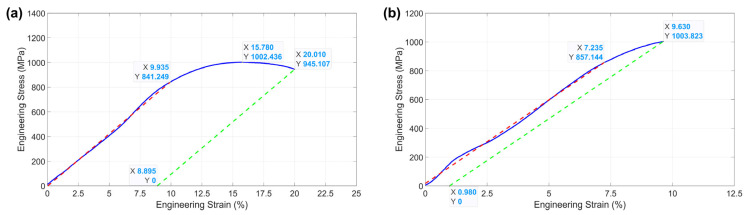
Two engineering stress vs. strain plots showing the response of (**a**) a relatively ductile dogbone 6 and (**b**) a brittle dogbone 15. The plastic strain is identified with an offset curve (dashed green) set at the fracture point that matches the slope of the elastic portion (dashed red). The compliance of the system is visible in the highly extended elastic portions of the plots.

While sample fracture was successfully constrained within the gauge section for all dogbones tested (Figure 6b,c and Supplementary File S1), a mixture of relatively ductile and brittle failure was recorded (Figure 8). The failure type was heavily dependent on the grain orientation within the gauge section. Brittle dogbones had grains oriented perpendicular to or at an angle off the loading direction. In the tests reported here, the six brittle dogbones generally had grains oriented straight up in a top-down SEM view or at a roughly 45° off the loading direction (Figure 6b). The three relatively ductile dogbones had grain orientations close to parallel to the loading direction.

### 4.1. Discussion—Sample Preparation

A balance must be struck between a lower FIB current that tends to have a sharper beam focus and a higher current that will cut quicker. Regardless of where that balance is for different materials or dogbone sizes, the taper reduction steps (Figure 5b,c) are critical as even small amounts can shift the measured mechanical response [15]. Additionally, notable taper renders the assumption of constant cross-sectional area invalid for transforming load–displacement to stress–strain.

The beam focus and beam drift in the PFIB were found to be the greatest contributors of difficulty in the dogbone polishing steps for this work. This was apparent when observing the collateral damage on the top surface of the dogbone head. Preferential milling of the softer Cu-rich phase occurred, leading to the formation of curtaining that propagated downwards into the gauge section. Beam drift caused the thin FIB polishing patterns to move away or into the free surface being polished. If the drift moved the pattern into empty space, time was wasted as the relative position of the pattern from the edge of the dogbone head was lost and must be located again with another sequence of polishing FIB patterns. If the drift moved the pattern into the dogbone head, the polishing pattern cuts the full depth of material instead of just the tapered gauge section. This exacerbates the taper of the gauge section as the sides of the dogbone head were quickly milled away, and the original relative position of the pattern from the edge was lost.

Depositing a rectangular platinum cap along the top and bottom edges of the dogbone head just prior to the steps in Figure 5c notably improved the surface polish quality in the PFIB but showed minimal improvement in the Ga+ FIB. However, the harder platinum caps assisted in accurate measurement of the thickness of the polished dogbone.

### 4.2. Discussion—Experiment

It was found that the PI 89 transducer consistently reported a small amount of “ghost” load values in the range of 0 to roughly 250 µN. Higher “ghost” load values were observed after large transducer position adjustments were made for the gripper but were easily tared out prior to starting each tensile pull. The residual “ghost” load leftover was not significant compared to the measured load values in both the elastic and/or plastic portions of the sample load curves and, thus, had negligible contribution to the stress–strain error.

It is the dogbone dimension measurements that contribute to the error in stress (gauge width and thickness) and strain (gauge length). Of these three measurements, gauge length and thickness are more difficult to measure accurately. The visual clarity of the gauge section’s two side edges allows for precise measurement of the gauge width, and significant error from this dimension is not expected.

Meanwhile, the inclusion of circular arcs joining the load surfaces to the gauge section makes it difficult to decern accurately the endpoints of the gauge length. However, the small radius used means that the error can be reasonably confined to roughly 0.5 µm. This confinement comes from the measurement procedure used. A rectangle measurement tool is used in the SEM and one of its long side edges is positioned to overlap with a side edge of the gauge section; the two corner arcs have a large curvature due to their small radii and quickly intersect the rectangle side edge.

Meanwhile, the difficulty in measuring dogbone thickness arises from collateral FIB damage to the side edges of the dogbone head during the final polishing step in Figure 5c. This will result in the dogbone head being slightly thinner than the thickness in the gauge section. This is especially notable in the dogbones that were polished in the PFIB whose beam focus quality is less than that of the Ga+ FIB. In general, the measurement error of dogbones polished with the Ga+ FIB and/or with Pt caps is roughly 100 nm, increasing to about 250 nm for those polished in the PFIB without a Pt cap. The thickness is determined in the SEM view by positioning a rectangle measurement tool such that its two opposite side edges overlap with the top and bottom edges of the dogbone; the error arises from repositioning the rectangle side edges so that they overlap with the slightly visible gauge section in the middle. However, it is important to remember that some of the gauge section visibility is due to the tapered surface as the low-current polishing step does not cut the surface straight at large depths.

A summary of the engineering stress–plastic strain values is tabulated in Table 1. Additional figures of stress vs. strain plots and SEM images of the dogbones tested are included in Supplementary File S1.

## 5. Finite Element Analysis—Dogbone Geometry

A thorough finite element analysis (FEA) mapping the von Mises stress distribution on varying dogbone geometries was performed.

This type of analysis was carried out previously by Casari et al. [14] on a similar micro-tensile experimental setup to identify an optimal dogbone geometry. The previous authors focused on the ratio of corner radius to gauge width to optimize their dogbone geometry. They mentioned that they also tested the effect of gauge length but provided no results on that part of their simulation efforts. This is most likely because they decided to fix the gauge length to 10 µm, constraining the dogbone length to 30 µm.

In this study, a couple of additional parameters on top of those already mentioned were tested. The list of tested parameters is as follows:

(1) Corner radius.
(2) Load surface angle.
(3) Load surface length.
(4) Dogbone head length.
(5) Gauge length.

Notably absent is the gauge width, which was fixed to 4 µm for a few reasons. The first is that, in this study, multi-phase nanostructures made by DED-LB were tested as opposed to single crystals. The microstructure was not fine enough that a thin gauge width would capture enough grains along the cross-section to be representative of the entire bulk sample. Conversely, the microstructure was not coarse enough that a single large grain could encompass the entire gauge section. Second, the difference in hardness of the two phases implies that there will be some surface roughness along the two parallel edges of the gauge section. This is a byproduct from polishing the sidewalls of the gauge section. When the gauge width is reduced, the relative ratio of the rough surface region width to the total gauge width increases. Finally, the gripper is cut from a diamond conical indenter tip using FIB milling. As such, increasing the width of the gauge section requires a disproportionately larger increase in the volume of material needed to be removed, both from the gripper as well as when making the dogbone samples.

Additionally, we elect to initially fix the gauge length to 10 μm instead of simulating all six values. We hypothesize that the gauge length has only a small effect on the stress distribution profile as the gauge section is geometrically uniform along this dimension. This reduces the number of geometries needing to be simulated, and, after the other four parameters are optimized, we will test the rest of the gauge lengths.

### 5.1. Dogbone Geometry Variables

**Figure 9:**
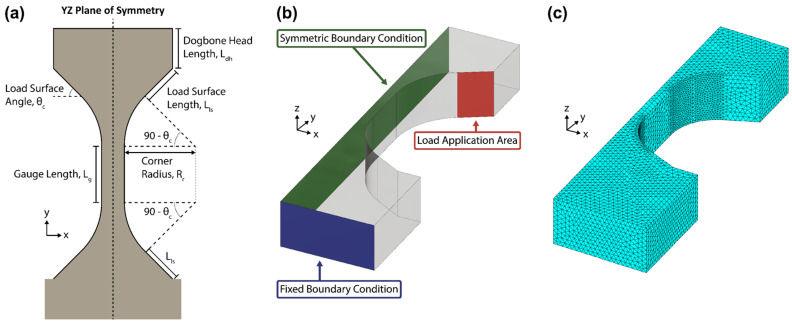
(**a**) Schematic of the dogbone template with the 5 adjustable parameters labeled. The arc-angle of the corner arc is the complement of the load surface angle to ensure its coincidence with the load surface length is smooth and continuous. (**b**) Diagram of a simulated dogbone geometry with the two boundary conditions and load application area highlighted. (**c**) An example of the meshed half-dogbone geometry. Visible is the refined mesh spacing along the curved regions.

Table 2 lists the first four parameters with ten values each and the gauge length with six values, of which only 10 µm was initially tested. Thus, ten thousand dogbone geometries were simulated. To reduce simulation runtime, only half of the dogbone geometries were drawn, meshed, and solved. This was possible because of the dogbones’ lengthwise symmetries as seen in Figure 9a. The last three parameters are the material properties, assumed to be isotropic, and the load, assuming no slippage.

**Table 2:** Dogbone testing parameters.

Variable	Symbol	Values	Units
Load surface angle	$\theta_{ls}$	0, 7.5, 15, 30, 37.5, 45, 52.5, 60, 67.5	degrees
Corner radius	$R_{c}$	2, 3, 4, 6, 8, 10, 12, 14, 16, 18	µm
Load surface length	$L_{ls}$	4, 5, 6, 7, 8, 9, 10, 11, 12, 13	µm
Dogbone head length	$L_{dh}$	4, 5, 6, 7, 8, 9, 10, 11, 12, 13	µm
Gauge length	$L_{g}$	4, 8, 10, 12, 16, 20	µm
Gauge width	$W_{g}$	4	µm
Dogbone thickness	$T_{d}$	10	µm
Young’s modulus	E	160	GPa
Poisson’s ratio	υ	0.3	1
Applied load	F	5000	µN

MATLAB’s partial differential equation toolbox was utilized, as it has a built-in structural analysis modeling function, “createpde”, for solving small-strain linear elasticity problems. The analysis type used was “static-solid”. The dogbone geometries were first constructed as a 2D shape using a collection of rectangles, triangles, and circles which were added and subtracted from each other using the “decsg” function. Then, any leftover lines inside the dogbone shape were removed using the “csgdel” function.

With this 2D shape, a 2D partial differential equation (PDE) model was first initialized in MATLAB to create a 2D geometry object. This 2D geometry object can then be extruded to the set dogbone thickness of 10 µm, becoming a 3D geometry object. Only then can the 3D PDE model be initialized and inserted in the 3D geometry object.

### 5.2. Mesh Convergence

With the 3D geometry object, a tetrahedral mesh, shown in Figure 9b, was created and the overall maximum mesh length was set to 1 $\left(H_{max}=1\right)$. The corner arc surfaces, defined by the corner radius and complement to the load surface angle, were selectively refined and the mesh spacing was set to 0.5 $\left(H_{refine}=0.5H_{max}\right)$. This refined mesh spacing was used as the highest gradients in stress concentration are expected along the shortest load transmittance path. The resulting meshes ranged from 39,397 to 108,190 elements for the smallest and largest dogbone geometries tested, respectively.

Prior to simulating the ten thousand geometries, the overall maximum mesh length was tested for convergence. A single dogbone geometry with the following parameters was created for this test: $\theta_{ls}=45$, $R_{c}=10$, $L_{ls}=10$, $L_{dh}=10$, $L_{g}=10$, $W_{g}=4$. Maximum mesh length values of 1.25, 1.0, 0.7, and 0.6 were used and the maximum stress value in the dogbone was recorded. The mesh convergence was defined as achieved when a doubling of the node density yielded less than 0.5% change in the calculated maximum stress. The 0.5% change criterion takes the absolute value of the difference in stress values. Near and at mesh convergence, the maximum stress value will tend to oscillate.

Compared to the initial mesh length value of 1.0, the node density is roughly doubled and tripled with a length value of 0.7 and 0.6, respectively. The corresponding change in maximum stress was 0.4% and 0.11%, respectively. Conversely, the node density is halved with a length value of 1.25 and the change in maximum stress was 0.54%. Thus, we confirm that our initial maximum mesh length of 1 meets the defined convergence criteria.

The benefit of using a larger mesh spacing, which still satisfies the convergence criteria, is the reduced runtime per iteration of the model. This is because while node density increases linearly, simulation runtime increases exponentially, roughly following $2^{n},$ where ***n*** is the ratio of the node densities. That is, a doubling or tripling of the node density results in the simulation runtime quadrupling or octupling, respectively.

All simulations were carried out on a desktop computer (AMD Ryzen Threadripper 3960X) and the 10,000-geometry test took roughly 3.5 days to complete.

### 5.3. Von Mises Stress Profile

**Figure 10:**
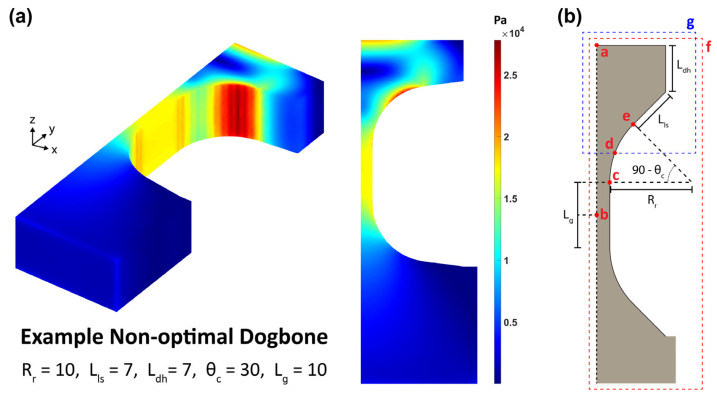
(**a**) The calculated von Mises stress profile of one dogbone, chosen as its non-optimal dimensions, produces clearly visible high-stress concentration regions outside the gauge section. (**b**) A schematic of the dogbone template with the 5 points and 2 regions sampled for calculating stress concentration ratios relative to the origin (point b). Point a is the top-center of the dogbone head. Point b is the center of the gauge. Point c is the intersection between the gauge and the corner arc. Point d is the midpoint of the circular arc. Point e is the intersection between the corner arc and the load surface. Region f encompasses the entire dogbone. Region g spans from the dogbone head to the midpoint of the circular arc.

Looking at the von Mises stress visualization in Figure 10a, we can see that there are three stress concentration regions: the top center of the dogbone head, along the corner arc, and a pair of narrow strips at the transition from the corner arc to the gauge section.

The first region arises when the dogbone head length is too small to diffuse the stress radiating from the load surfaces. In general, a lower load surface angle requires a larger dogbone head length. The third region is unavoidable as it is a consequence of the curvature stopping at the straight gauge section sides. Notably, this phenomenon appears even in the tangential tensile testing simulations conducted by Malhaire et al. [16] and Yang et al. [17]. However, the correct set of parameter dimensions can reduce the degree of stress concentration, as shown later in this work. Finally, the high stress of the second region is expected due to the transmission of load which is applied non-uniformly only on the two load surfaces. The opposing force from the substrate is uniform and thus we do not see the same stress band along the corner arc on the bottom half of the dogbone.

### 5.4. Stress Concentration Ratio

To simplify the von Mises stress analysis for identifying the optimal set of dimensions, a maximum stress concentration ratio $K_{c}$ was defined (Equation (1)) as the quotient of the stress values at the sampling points and regions defined in Figure 10b. For each sampling location, the maximum stress value through the entire dogbone thickness was used (Equation (2)).

(1) $$K_{c}=\max\left(\frac{\sigma_{\mathrm{vector}}}{\sigma_{\mathrm{origin}}}\right)$$

(2) $$\sigma_{\mathrm{vector}}=\max_{z}\left[\begin{array}{ccccccc} \sigma_{a}\;, & \sigma_{b}\;, & \sigma_{c}\;, & \sigma_{d}\;, & \sigma_{e}\;, & \sigma_{f}\;, & \sigma_{g} \end{array}\;\right],\;\mathrm{where}\;\sigma_{b}=\sigma_{\mathrm{origin}}$$

**Figure 11:**
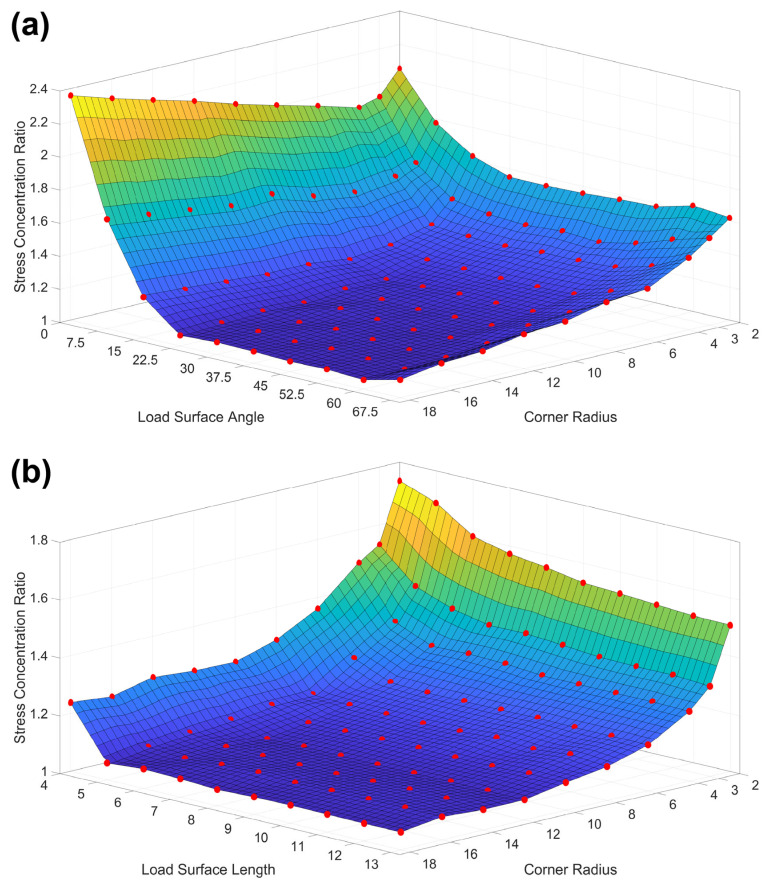

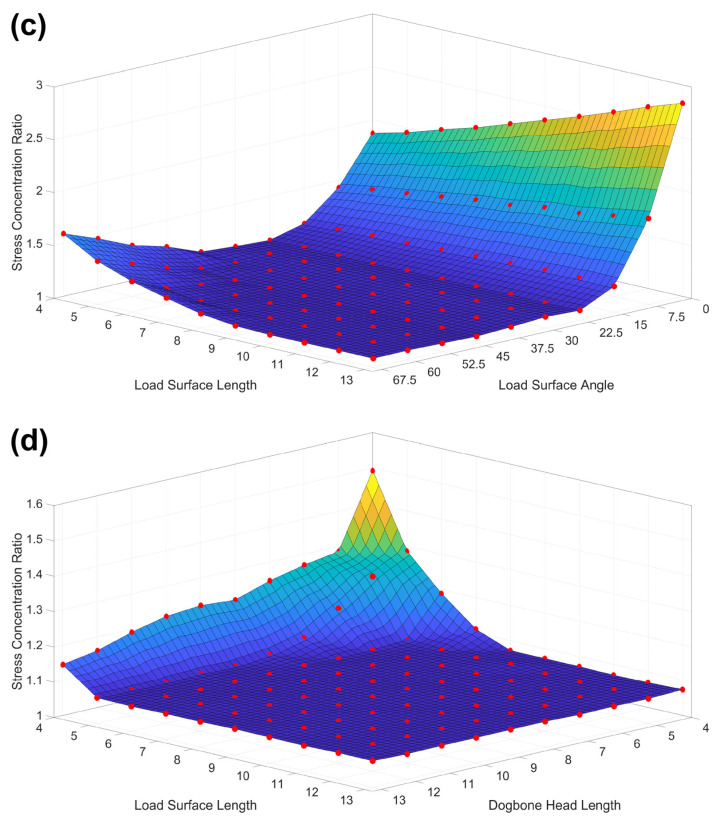
The four 3D surface plots of the stress concentration ratio as a function of two parameters. The 100 red dots on each plot represent the calculated ratio for a specific dogbone geometry and the surface is created using linear fit interpolation. For (**a**), the load surface length and dogbone head length are both fixed to 7 μm. For (**b**), the load surface angle and dogbone head length are fixed to 45° and 7 μm, respectively. For (**c**), the corner radius and dogbone head length are fixed to 12 μm and 7 μm, respectively. For (**d**), the load surface angle and corner radius are fixed to 45° and 12 μm, respectively.

The $K_{c}$ values were plotted as a function of two variable parameters while fixing the other two. This produces a three-dimensional (3D) surface plot which shows which combinations of values from those two parameters produce the lowest $K_{c}$ values. Figure 11 shows four out of the six unique two-parameter combinations plotted.

Intuitively, it was recognized that three variables can be increased indefinitely to yield lower stress concentration ratios: corner radius, load surface length, and dogbone head length. At the same time, it is expected that the rate of change in lowered $K_{c}$ values from increasing these same three variables will inevitably decrease in an exponential manner. Thus, the 3D surface plots will allow us to identify regions where further increases in certain parameters are no longer meaningful.

Additionally, micro-tensile dogbones are more time consuming to mill using an FIB compared to larger-scale tensile samples machined by vertical mills or electric discharge machining. Those same three parameters all contribute to increasing the overall length of the dogbone and, consequently, the time required to remove the taper along the top and bottom surfaces of the dogbone.

### 5.5. Influence of Corner Radius and Load Surface Angle

We can see that the first two-parameter plot (Figure 11a) has a couple of notable features. First, the high-stress concentration at a load surface angle of 0° indicates that a T-shaped dogbone cannot be an optimal geometry regardless of the values of the other parameters. This is corroborated by Du et al. [18], who also conducted finite element modeling (FEM) and testing of T-shaped dogbones. Second, it was observed that the stress concentration ratio rises beyond a load surface angle of 60°, forming a valley where the optimal combination of corner radius and load surface angle can be found. When comparative plots were made using load surface lengths larger than the fixed value of 7 μm here, the valley appears to open up at high corner radii and load surface angles. However, it is very likely that if load surface angles greater than 67.5° were tested, the stress concentration ratio would rise again, and that the optimal region would still fall within the 15° to 60° range. This is because as the load surface angle approaches 90°, the assumption of no slippage no longer holds true.

For the corner radius, it is observed that for load surface angles greater than 22.5°, the stress concentration ratio decreases continuously with larger values. Interestingly, for small load surface angles that are normally not optimal, it is preferable to choose a smaller corner radius as there is a small dip in the stress concentration ratio for corner radii 2 to 8, bracketing a minimum at around 4. Interestingly enough, Xu et al. [5] selected a radius of 3 μm, close to the optimal value, for their T-shaped dogbones and acquired reasonable agreement in measured strength values between their micro- and macro-scale tests. Thus, while their dogbone geometry was not optimal from a stress concentration ratio standpoint, it does not appear to be the sole limiting condition to acquiring some good mechanical property measurements. Indeed, we observed that our experimentally tested dogbones all had acceptable fractures in the middle of the gauge despite having a high-stress concentration ratio of about 1.40.

### 5.6. Influence of Load Surface Length and Dogbone Head Length

In Figure 11b,d, it is noted that the load surface length has a distinct lower bound of 5 μm, below which the stress concentration ratio increases significantly. Meanwhile, Figure 11c shows that the load surface length has a large optimal valley, like Figure 11a, except with a higher lower bound of acceptable load surface angles starting at 22.5° instead of 15°. Additionally, at low load surface length values, the upper bound of acceptable load surface angles also decreases from 60° in Figure 11a to just 45° at 6 μm. Since dogbones with larger dimensions are not efficient to make with FIB milling, the true range of optimal load surface angles will fall within this small range.

As shown in Figure 10a, high-stress values can arise at the midpoint of the top edge of the dogbone head when the dogbone head length is set too small. Notably, this also results in a concurrent increase in the stress along the corner arc. Thus, the dogbone head length, which is often selected arbitrarily, plays an important role in ensuring that stress outside of the gauge section is reduced to an acceptable amount. Figure 11d shows that dogbone head length has an inverse relationship with the load surface length. For smaller load surface angles, it was found that the minimum acceptable dogbone head length increased for fixed values of the other two parameters.

## 6. Identification of Optimal Geometry

An optimal dogbone geometry must satisfy two criteria, balancing theory and practicality. The FEA shows that three of the variable parameters have no upper limit to being beneficial in reducing the stress concentration ratio. However, micro-tensile dogbones are constrained by the high FIB milling time required to manufacture each sample along with the gripper. Additionally, large depths are difficult to polish using an FIB without leaving behind a noticeable taper that deviates the rectangular cross-section assumption used to calculate the stress–strain response from the force–displacement data. While higher FIB currents can reduce the taper, they tend to produce a worse surface polish and will create a thicker layer of amorphized material.

The following constraints were set:

(1) A dogbone head width of no more than 25 µm (Equation (3)).
(2) A dogbone length of no more than 30 µm; length is calculated from the top edge of the dogbone head to the bottom of the gauge section (Equation (4)).

The first constraint is due to the conical indenter tip that the gripper is manufactured from. The sides have an angle of roughly 30° from the vertical, and thus, a wider dogbone head requires a much wider base cut in the gripper. This can present clearance problems when the gripper is moved towards the dogbone. Additional material along the edges beside the dogbone must be milled away using an FIB to ensure the wide base of the gripper does not have an impact.

(3) $$25\geq W_{dh}=2\left[W_{g}+R_{c}\cos\left(90-\theta_{ls}\right)+L_{ls}\cos\left(\theta_{ls}\right)\right]$$

(4) $$30\geq L_{dl}=\left[L_{g}+R_{c}\sin\left(90-\theta_{ls}\right)+L_{ls}\sin\left(\theta_{ls}\right)+L_{dh}\right]$$

With these two equations, a logical mask was created to identify the dogbone geometries that satisfy both conditions. Additionally, a third condition was implemented by fixing the load surface angle. While the FEA simulations tested angles in increments of 7.5°, analysis showed that (i) the optimal load surface angle range spans a small range from 22.5° to 45°, and (ii) the stress concentration ratios within that optimal angle range had low variance. Thus, by fixing the angle to a value within its optimal range, the optimized values of the other three parameters of corner radius, load surface length, and dogbone head length can be more easily identified since the search space is reduced from 4D to 3D.

The analysis was focused on two angles, specifically 30° and 45°.

(1) When the load surface angle was fixed to 45°, the optimal dimensions were as follows:
  - Corner radius = 12 µm.
  - Load surface length = 6 µm.
  - Dogbone head length = 7 µm.
  - Peak stress concentration ratio = 1.0704.
(2) When the load surface angle was fixed to 30°, the optimal dimensions were as follows:
  - Corner radius = 10 µm.
  - Load surface length = 6 µm.
  - Dogbone head length = 8 µm.
  - Peak stress concentration ratio = 1.0946.

As expected, when the load surface angle is 30°, the dogbone geometries reach the width constraint first compared to 45° and vice versa regarding the height constraint.

In general, all the dogbone geometries exhibited a band of high stress at the intersection between the gauge section and the corner arc. This is understandable as the stress transmittance following the corner arc stops its curved path at the start of the straight gauge. The issue is that this band of high stress can drown out stress values at other regions such as the intersection between the end of the corner arc and the load surface, or at the top center of the dogbone head.

**Figure 12:**
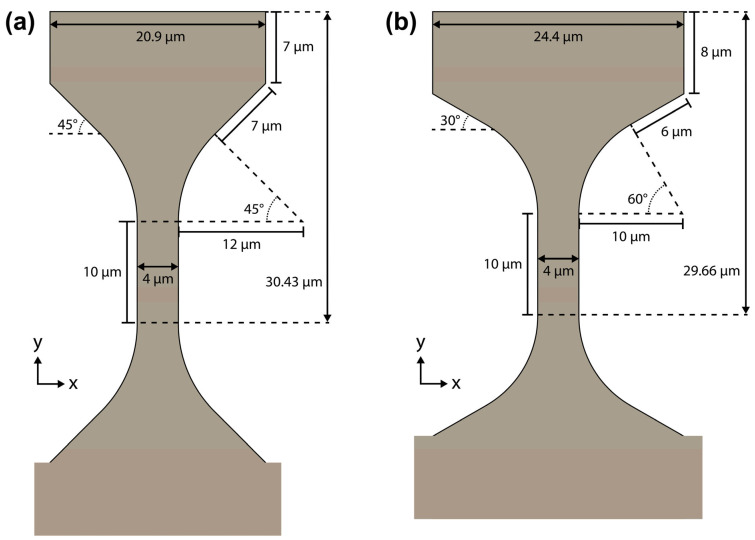
Schematics of the optimal dogbone geometries with (**a**) 45° and (**b**) 30° load surface angles when the width and height are constrained to no more than 25 µm and ~30 µm, respectively. The 45° geometry has a height of 30.43 µm which was found to yield a better von Mises stress profile for a minimal length increase above the original 30 µm constraint.

**Figure 13:**
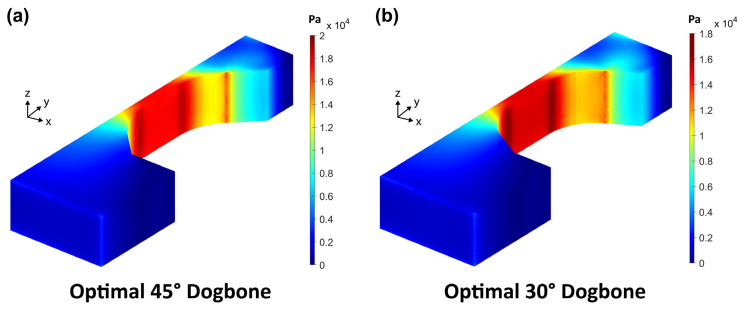
The corresponding von Mises stress profiles of the (**a**) 45° and (**b**) 30° optimal geometries. Note the different color bar scales. In both geometries, the red vertical stress bands at the intersection of the corner arc and load surface have stress values lower than the center of the gauge.

Therefore, a second logical test case was investigated where the high-stress band was excluded. For this test case, only the nodes that lie from the middle of the corner arc to the top of the dogbone were sampled (Equations (5) and (6)). Then, the stress concentration values were run through the same three logical masks as the first logical test case (width constraint, height constraint, and fixed angle). This second logical test case confirms that the optimal geometry for the 30° load surface angle case remains the same (Figure 12b and Figure 13b). The peak stress concentration ratio is 1.0946 with the high-stress band included and 1.0 when it is excluded.

(5) $$\sigma_{{\mathrm{vector}}_{\mathrm{ex}}}=\left[\begin{array}{ccccc} \sigma_{a}\;, & \sigma_{b}\;, & \sigma_{d}\;, & \sigma_{e}\;, & \sigma_{g} \end{array}\;\right],\;\mathrm{where}\;\sigma_{b}=\sigma_{\mathrm{origin}}$$

(6) $$K_{c_{\mathrm{ex}}}=\max\left(\frac{\sigma_{{\mathrm{vector}}_{\mathrm{ex}}}}{\sigma_{\mathrm{origin}}}\right)$$

However, for the 45° load surface angle case, a slight modification of the dimensions produces a minutely improved stress distribution that has a reduced stress band at the intersection between the top of the corner arc and the load surface. Here, the load surface length = 7 µm, and the dogbone head length = 6 µm. The peak stress concentration ratio is 1.0722 with the high-stress band included and 1.0 when it is excluded. When compared to the old optimal geometry, its peak stress concentration ratio excluding the high-stress band was 1.0043. If a small amount of tolerance was allowed in the height constraint, a further improvement could be attained with a load surface length = 7 µm and a dogbone head length = 7 µm. This geometry has a height of 30.43 µm and its peak stress concentration ratio is 1.0732 with the high-stress band included and 1.0 when it is excluded (Figure 12a and Figure 13a).

### Influence of Gauge Length

With the two optimal dogbone geometries’ parameters defined, the influence of the gauge length was analyzed. This parameter was previously fixed to 10 μm as it was expected to have a relatively small influence on the stress concentration ratio, even across a large range of values.

**Figure 14:**
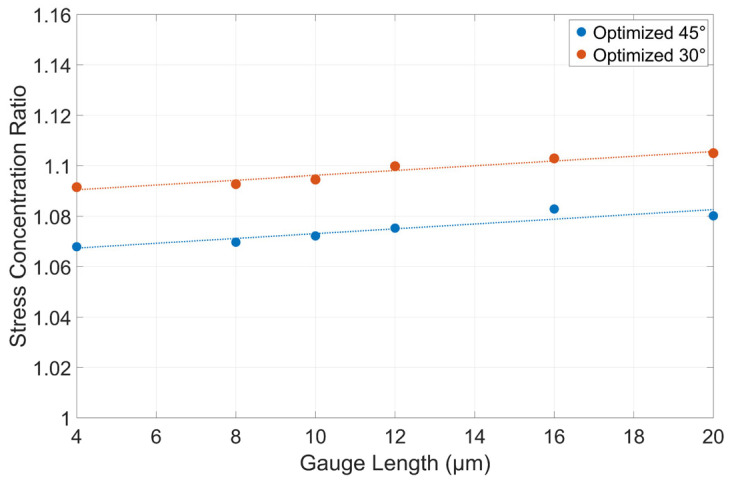
The stress concentration ratios of the 45° and 30° optimal geometries testing the other five gauge lengths (4, 8, 12, 16, 20 µm) alongside the 10 µm. Relative to the fixed gauge width of 4 µm, these represent length-to-width aspect ratios of 1, 2, 2.5, 3, 4, and 5, respectively. The dotted lines are linear fit curves.

In Figure 14, it is observed that the linear fit line shows a small upwards trend in stress concentration. However, the values of interest are 4, 8, and 10 μm, as larger gauge lengths present a challenge when removing the taper in FIB. For both the 30° and 45° geometries, the decrease in stress concentration ratio from 10 μm to 4 μm was less than one percent. Thus, while it is theoretically optimal to choose a smaller gauge length than the 10 μm used initially, this decision should be made more so to improve the taper reduction in the dogbone surfaces. Additionally, very small gauge lengths below 6 μm are likely not achievable, as they would require very slim gripper jaws, increasing the propensity that they fracture during loading.

## 7. Conclusions

A detailed and time-efficient procedure for FIB milling and testing uniaxial tensile dogbone samples was created for the Bruker PI 89 PicoIndenter. The methodology is also applicable to earlier iterations of Bruker’s PI80 series of nanomechanical testers.

It was found that alignment markers on both the sample SEM pin stub and 45° holder (if used) are crucial in preventing misalignment during FIB milling of the dogbone samples and when mounting the pin stub onto the PI 89’s stage. Additionally, the usage of bitmap patterns simplifies the FIB process by combining multiple manually placed FIB patterns into one. Finally, the FIB patterning was specifically ordered to reduce the amount of ion implantation into the polished top and bottom surfaces of the dogbone (yz-view). Our final polishing step ensures ion implantation is concentrated in the top of the dogbone head, which is a non-loadbearing region.

The procedure was tested with nine dogbones made from a Fe-45Cu bulk sample manufactured by powder DED-LB. In all dogbones, fracture was successfully confined within the gauge section and biased towards the middle. In all cases, the fracture type, brittle (<1% plastic strain to failure) vs. relatively ductile (>8% plastic strain to failure), was entirely dependent on the grain orientation. Dogbones with grains oriented perpendicular or at a large angle off the loading direction experienced brittle fracture and vice versa. Most of the dogbones tested had an engineering ultimate tensile strength ~1000 MPa regardless of their fracture type. Meanwhile, the engineering yield strengths had a large amount of variance with a median of about 640 MPa.

An extensive FEA study was carried out to optimize the dogbone geometry by defining a general dogbone shape as a function of six adjustable dimensions (parameters). Ten thousand geometries were tested, and for each, a stress concentration ratio was defined by the quotient of the maximum stress and the stress at the center of the gauge section. Comparisons of the stress concentration ratios showcased the influence of each individual parameter, of which the corner radius and load surface angle had the greatest detrimental effect when set outside their optimal ranges. The dogbone head length was surprisingly also an important dimension that could result in a very high-stress concentration at the top of the dogbone when set too small, an effect observed mainly when the load surface angle and load surface length are both set towards the lower end of our tested parameter ranges.

Finally, two sets of optimal micro-tensile dogbone geometries were identified, at 30° and 45° load surface angles, where the width and length are constrained to 25 µm and roughly 30 µm, respectively. The stress concentration ratios for the two were 1.0946 and 1.0732, respectively. The influence of gauge length was tested at the end using these two optimal geometries, and it was determined that smaller gauge lengths only minutely reduced the stress concentration ratio. A smaller gauge length is also preferable as it reduces the difficulty of the FIB taper reduction step. However, the actual gauge length chosen will be dictated by the minimum allowable size of the gripper jaws, which must be large enough to avoid fracturing themselves during testing. 

## Figures and Tables

**Table 1:** Dogbone tensile results (engineering stress vs. strain).

Dogbone	Grain Orientation Relative to Loading	Yield Strength (MPa)	Ultimate Strength (MPa)	Plastic Strain (%)
6	parallel	831 ± 10	990 ± 12	8.8 ± 0.2
7	parallel	630 ± 11	928 ± 16	8.8 ± 0.2
8	parallel	613 ± 10	934 ± 15	11.7 ± 0.3
9	perpendicular	566 ± 16	722 ± 20	0.9 ± 0
10	perpendicular	555 ± 16	789 ± 23	2.0 ± 0.1
11	perpendicular	1007 ± 8	1063 ± 8	3.1 ± 0.1
12	perpendicular	597 ± 4	996 ± 7	1.7 ± 0.1
15	perpendicular	850 ± 7	995 ± 8	1.0 ± 0
16	perpendicular	614 ± 5	842 ± 6	negligible

The two plastic strain error bounds of ±0 for dogbones 9 and 15 are a product of the calculated error being less than 0.05. Dogbones 1 to 5 are excluded since they were made without corner arcs and had fractures close to the dogbone head. Dogbones 13 and 14 are excluded as an error was made when applying the FIB pattern rotational alignment correction described in Section 2.3.

## Data Availability

The raw data supporting the conclusions of this article will be made available by the corresponding author on request.

## Supplementary Materials

The following supporting information can be downloaded at https://www.mdpi.com/article/10.3390/ma17215144/s1, one Supplementary File S1 containing the stress vs. strain plots and SEM images of dogbones not shown in the main article.
