# Supplementary material for: Optimal Geometry for Focused Ion Beam-Milled Samples for Direct-Pull Micro-Tensile Testing Performed In Situ in a Scanning Electron Microscope
Source: Materials (Basel). 2024 Oct 22;17(21):5144. doi: 10.3390/ma17215144 (PMC11547548; doi:10.3390/ma17215144)
Supplement: Supplementary file 1 [file materials-17-05144-s001.zip › materials-3247813-supplementary.pdf]

Supplementary File S1

Dogbone 6

The engineering stress vs. strain plot is omitted here as it is included in **Section 4.**, **Figure 8a** of the paper.

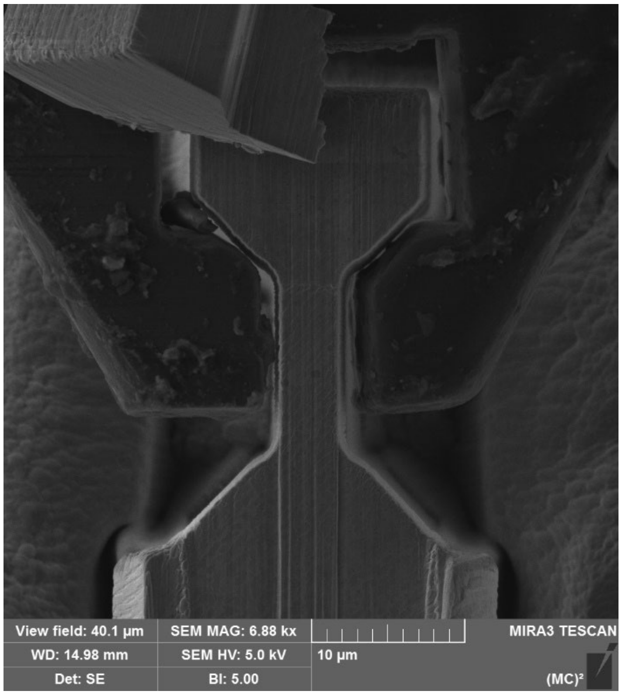

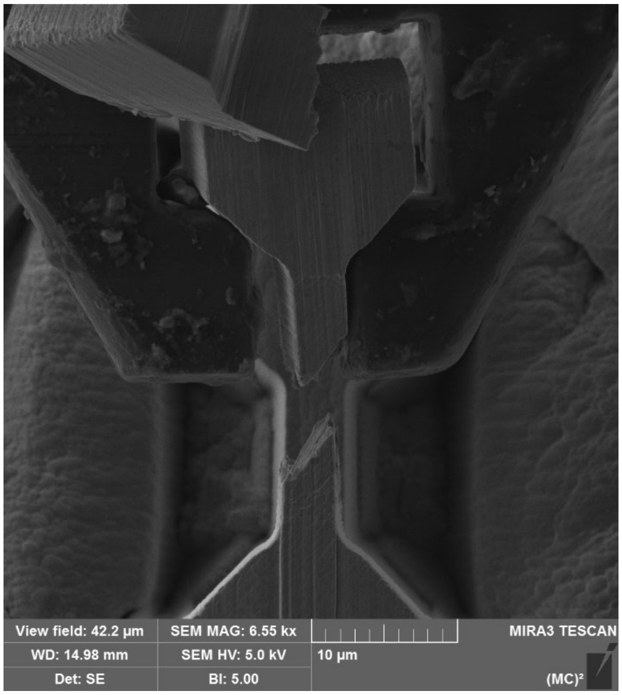

On the left is a SEM image of the dogbone aligned inside the gripper. On the right is another image of the dogbone post-fracture.

For this test, the broken dogbone head of dogbone 5 is visible, adhered to the top surface of the gripper due to electrostatic forces. That dogbone head was pushed out of the gripper using the pillar method described in **Section 3.2.** of the paper and does not contact dogbone 6 during its tension test.

Dogbone 7

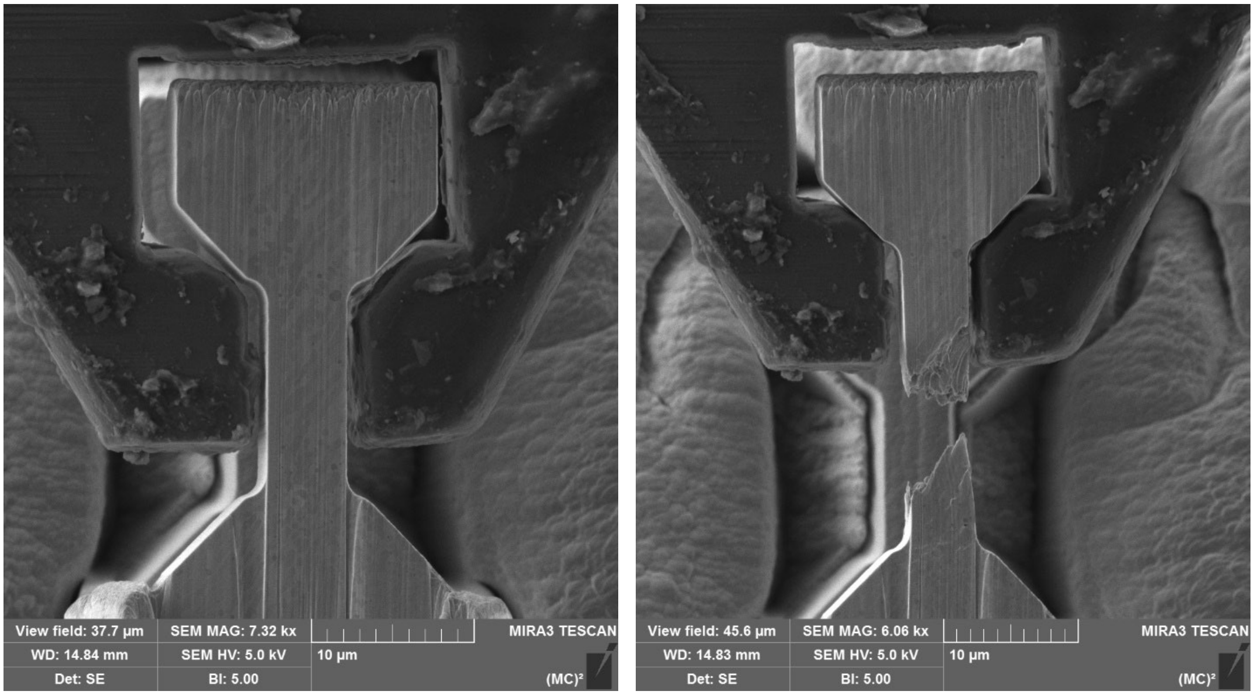

On the left is a SEM image of the dogbone aligned inside the gripper. On the right is another image of the dogbone post-fracture.

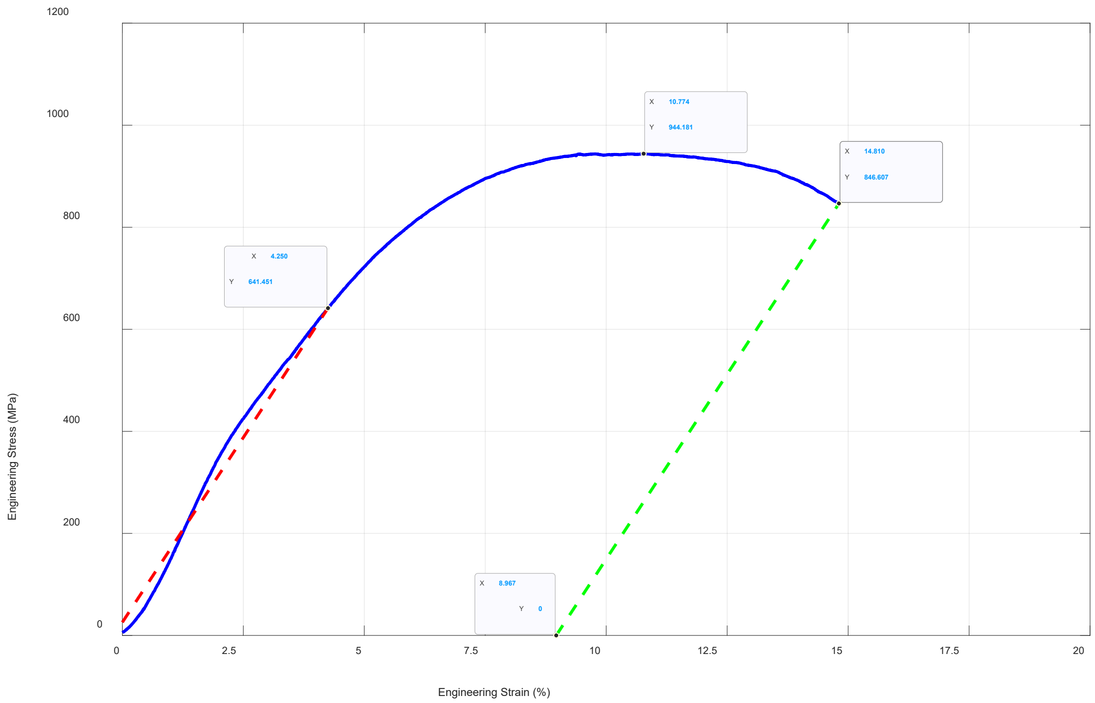

The plot of the engineering stress vs. strain. The plastic strain is identified with an offset curve (dashed green) set at the fracture point that matches the slope of the elastic portion (dashed red).

Dogbone 8

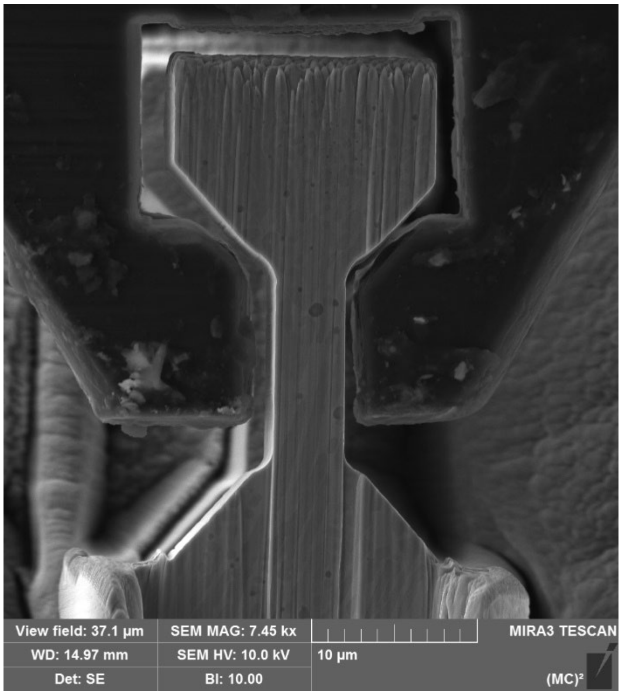

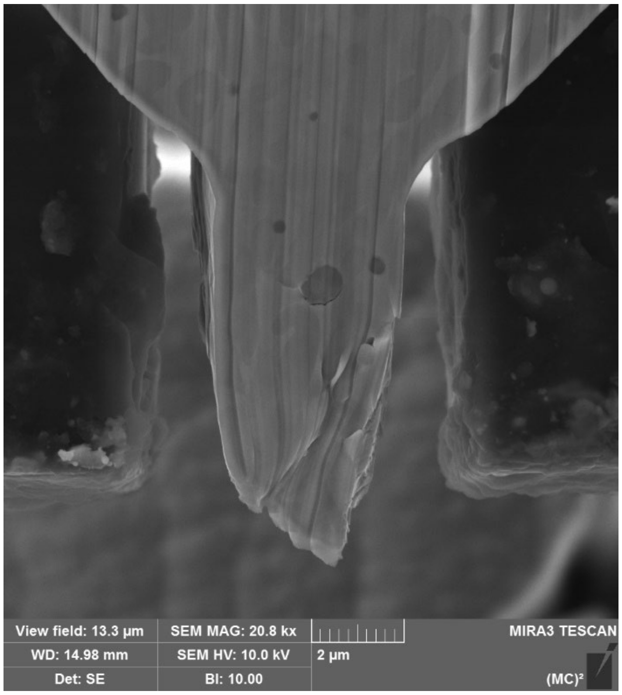

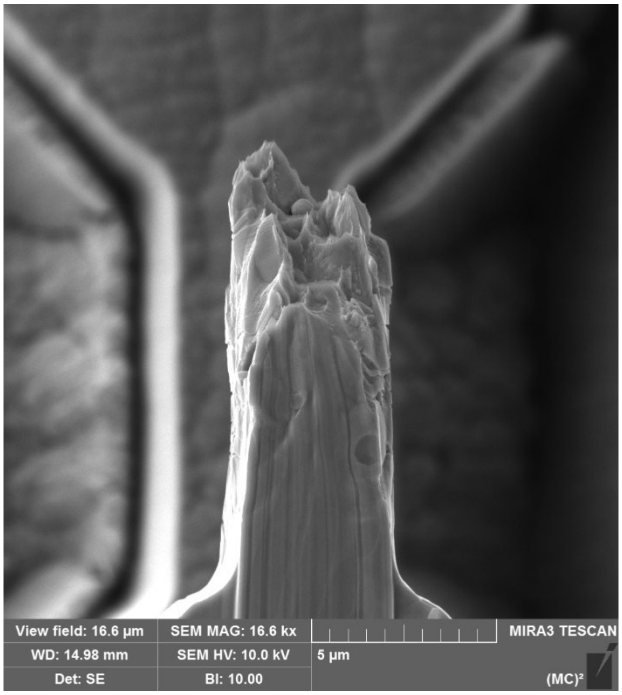

The first image is a SEM image of the dogbone aligned inside the gripper. The second and third images are of the dogbone post-fracture.

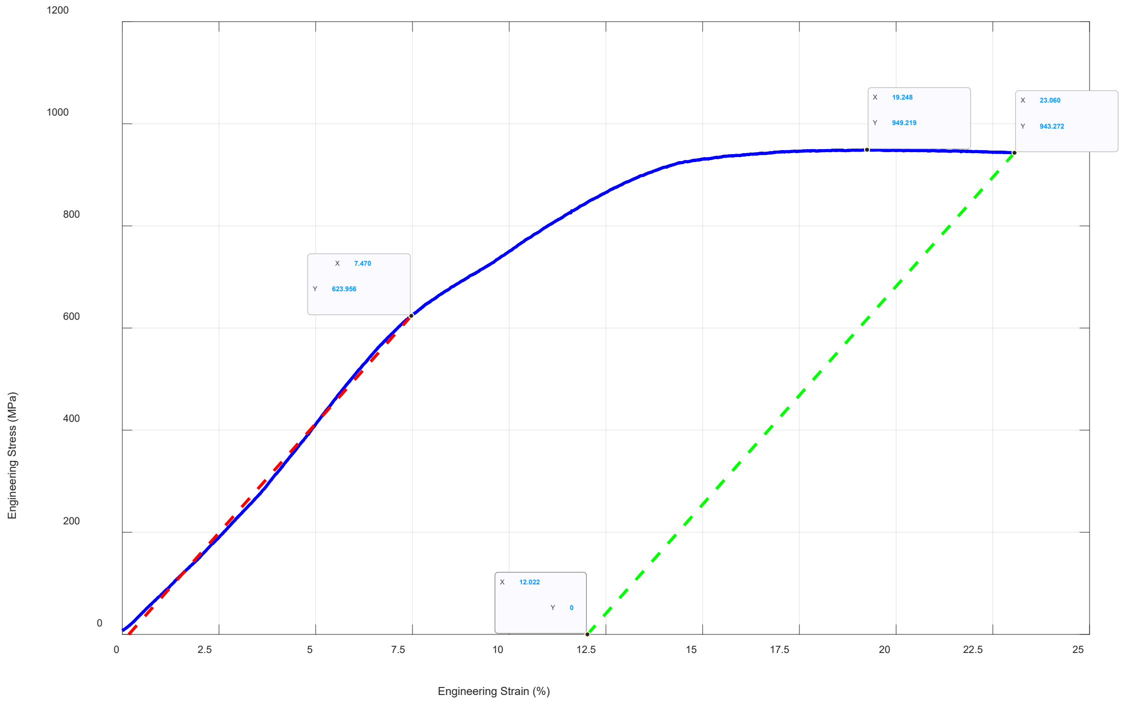

The plot of the engineering stress vs. strain. The plastic strain is identified with an offset curve (dashed green) set at the fracture point that matches the slope of the elastic portion (dashed red).

Dogbone 9

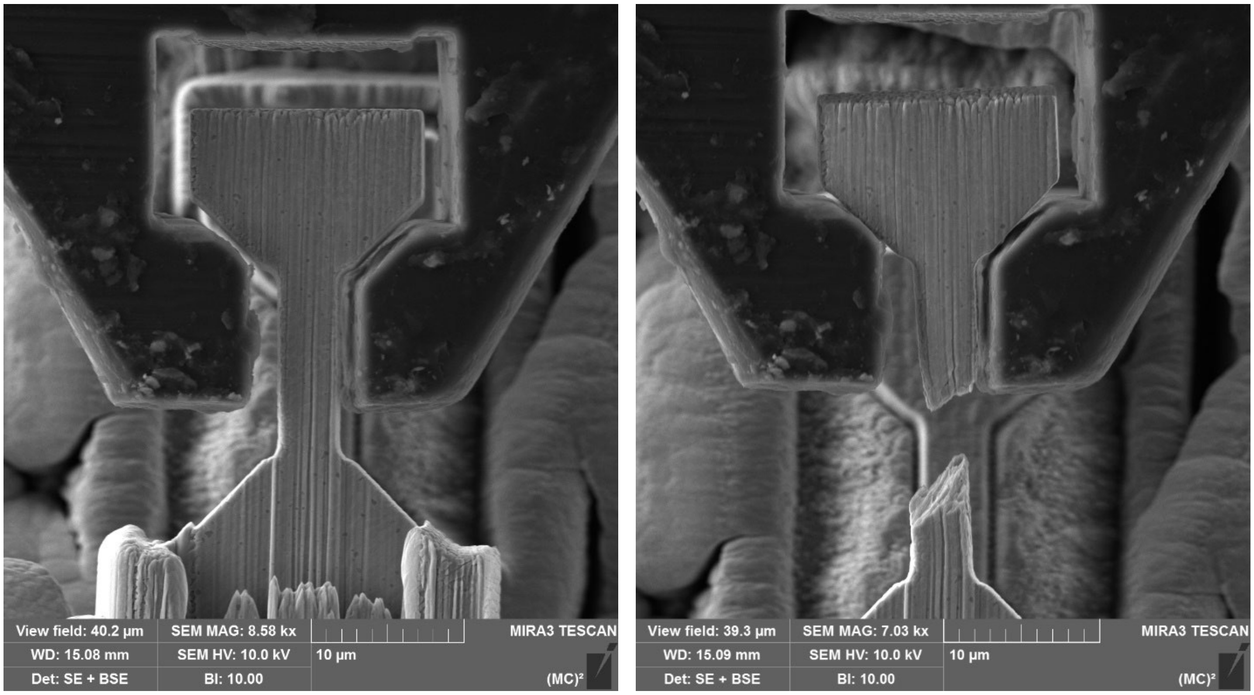

On the left is a SEM image of the dogbone aligned inside the gripper. On the right is another image of the dogbone post-fracture.

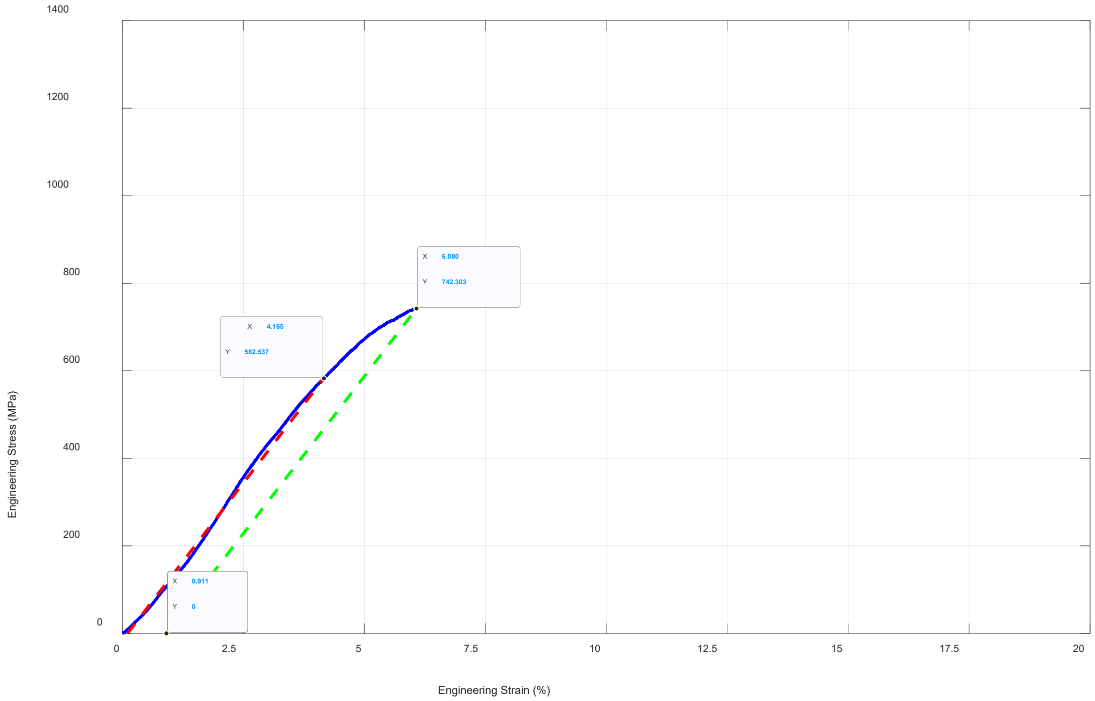

The plot of the engineering stress vs. strain. The plastic strain is identified with an offset curve (dashed green) set at the fracture point that matches the slope of the elastic portion (dashed red).

Dogbone 10

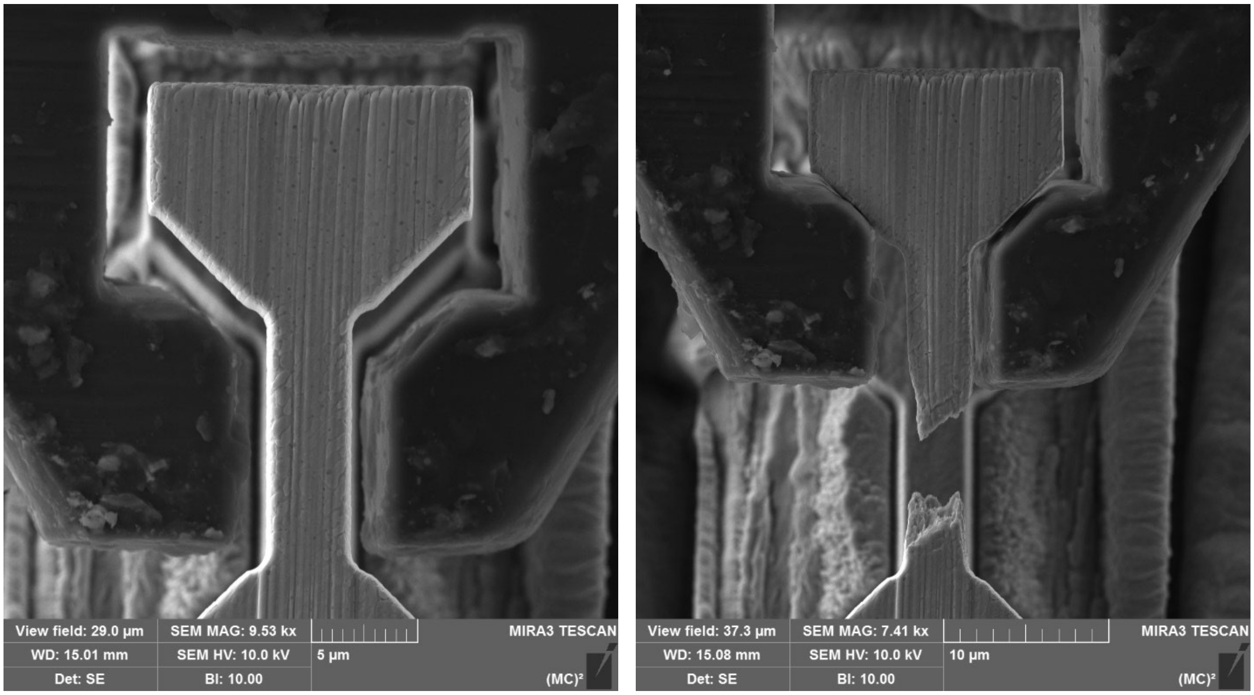

On the left is a SEM image of the dogbone aligned inside the gripper. On the right is another image of the dogbone post-fracture.

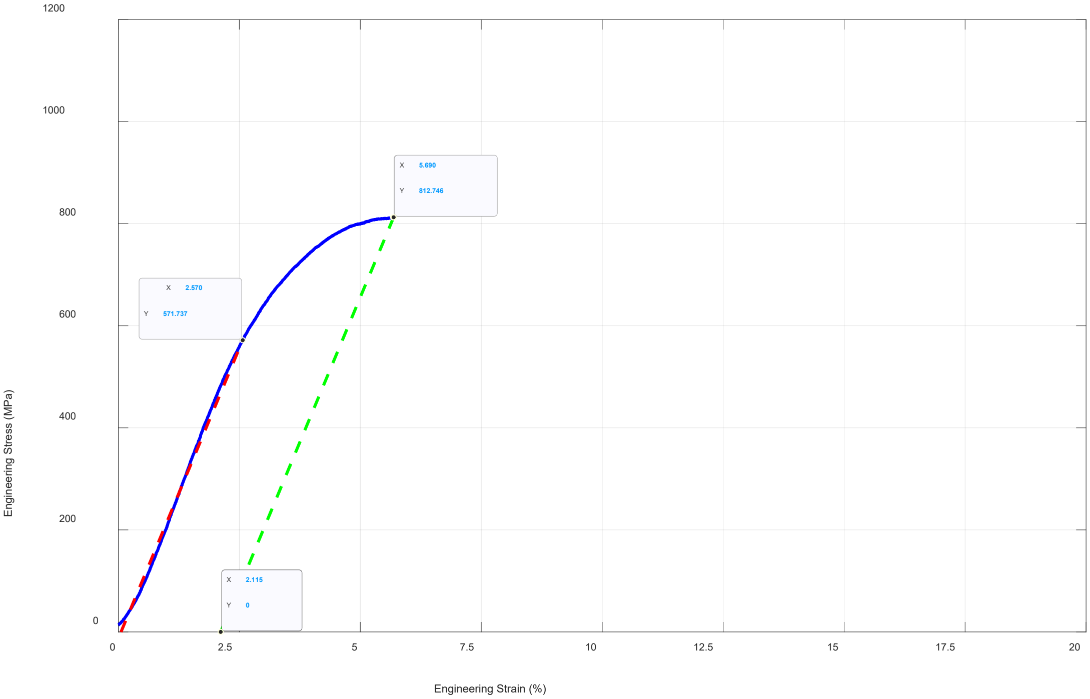

The plot of the engineering stress vs. strain. The plastic strain is identified with an offset curve (dashed green) set at the fracture point that matches the slope of the elastic portion (dashed red).

Dogbone 11

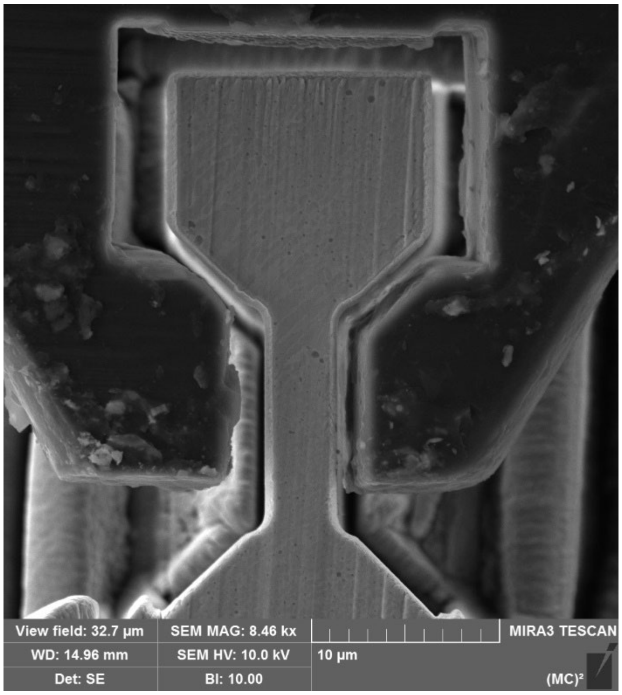

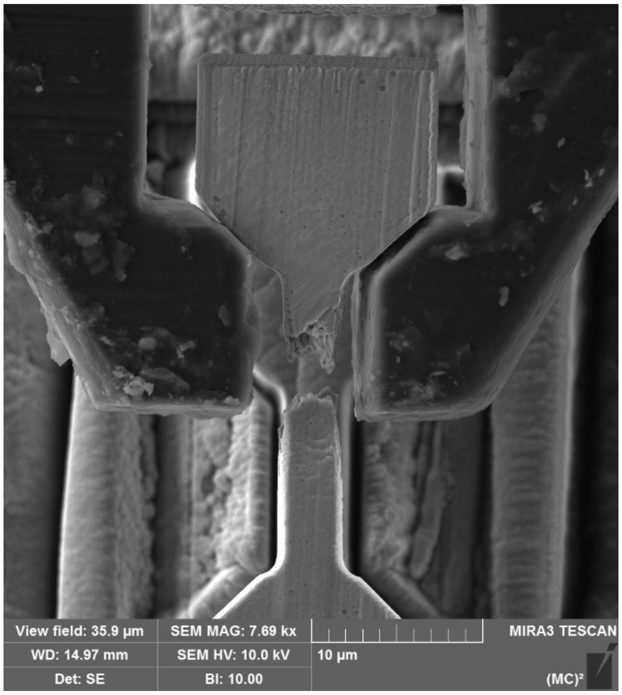

On the left is a SEM image of the dogbone aligned inside the gripper. On the right is another image of the dogbone post-fracture.

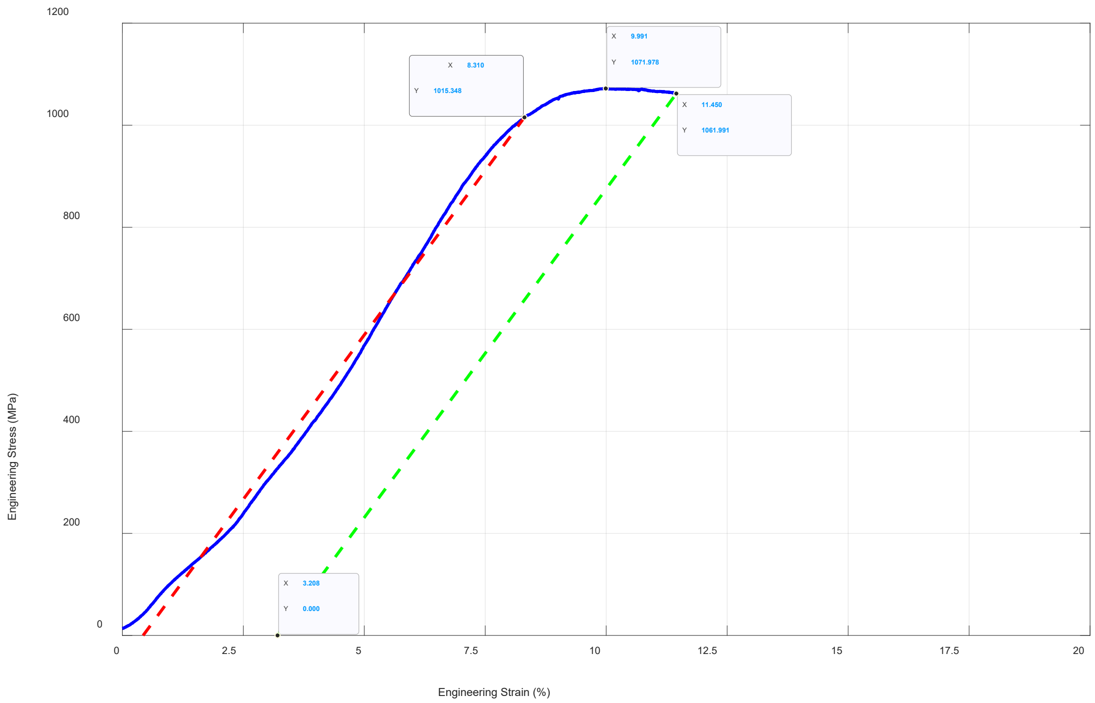

The plot of the engineering stress vs. strain. The plastic strain is identified with an offset curve (dashed green) set at the fracture point that matches the slope of the elastic portion (dashed red).

Dogbone 12

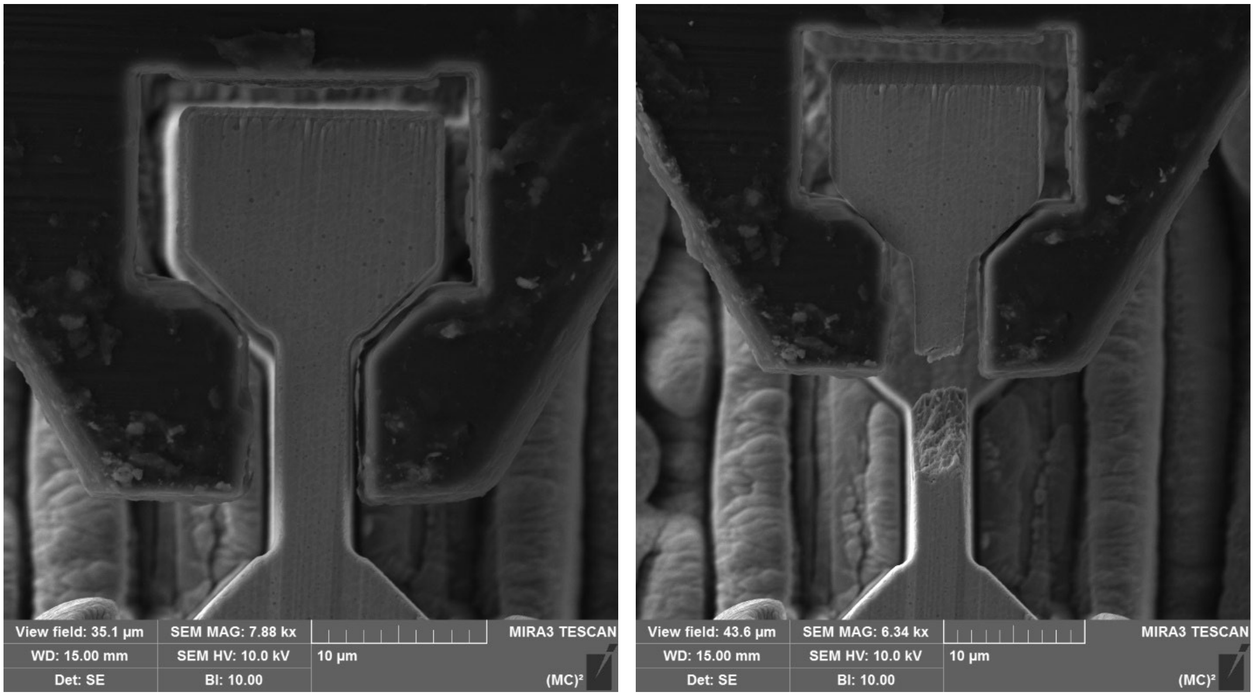

On the left is a SEM image of the dogbone aligned inside the gripper. On the right is another image of the dogbone post-fracture.

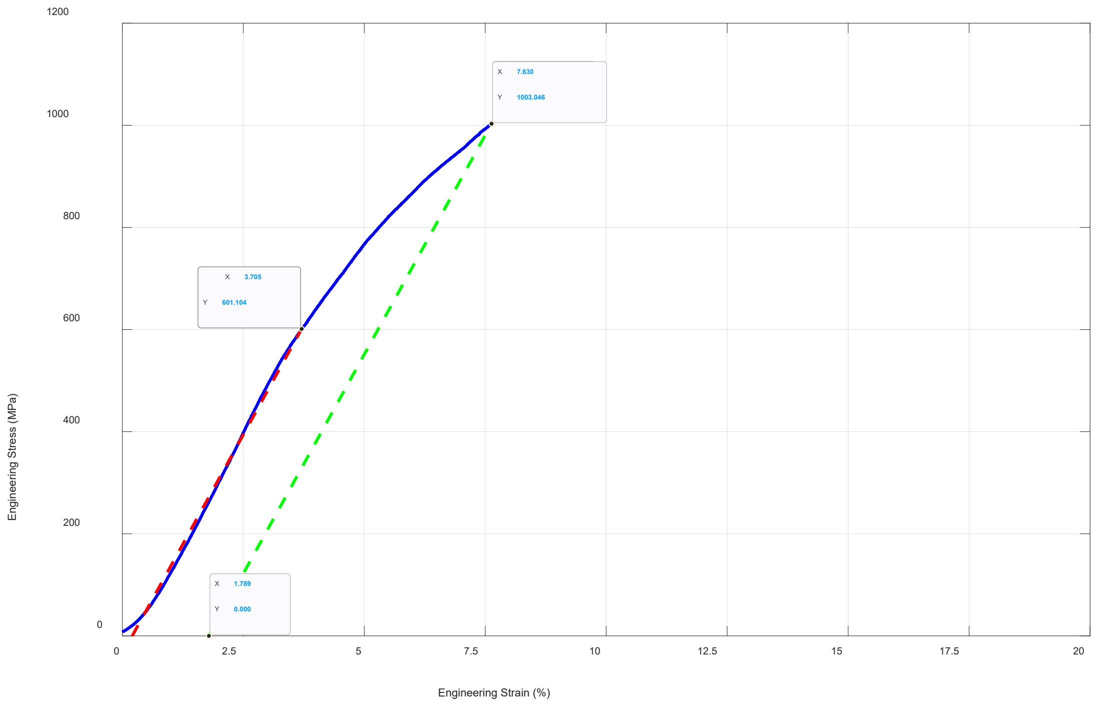

The plot of the engineering stress vs. strain. The plastic strain is identified with an offset curve (dashed green) set at the fracture point that matches the slope of the elastic portion (dashed red).

Dogbone 15

The engineering stress vs. strain plot and SEM images of the dogbone pre and post tensile testing are already included in the main paper in **Section 4.**, **Figure 8b** and **Section 3.**, **Figure 6b,c**, respectively.

Dogbone 16

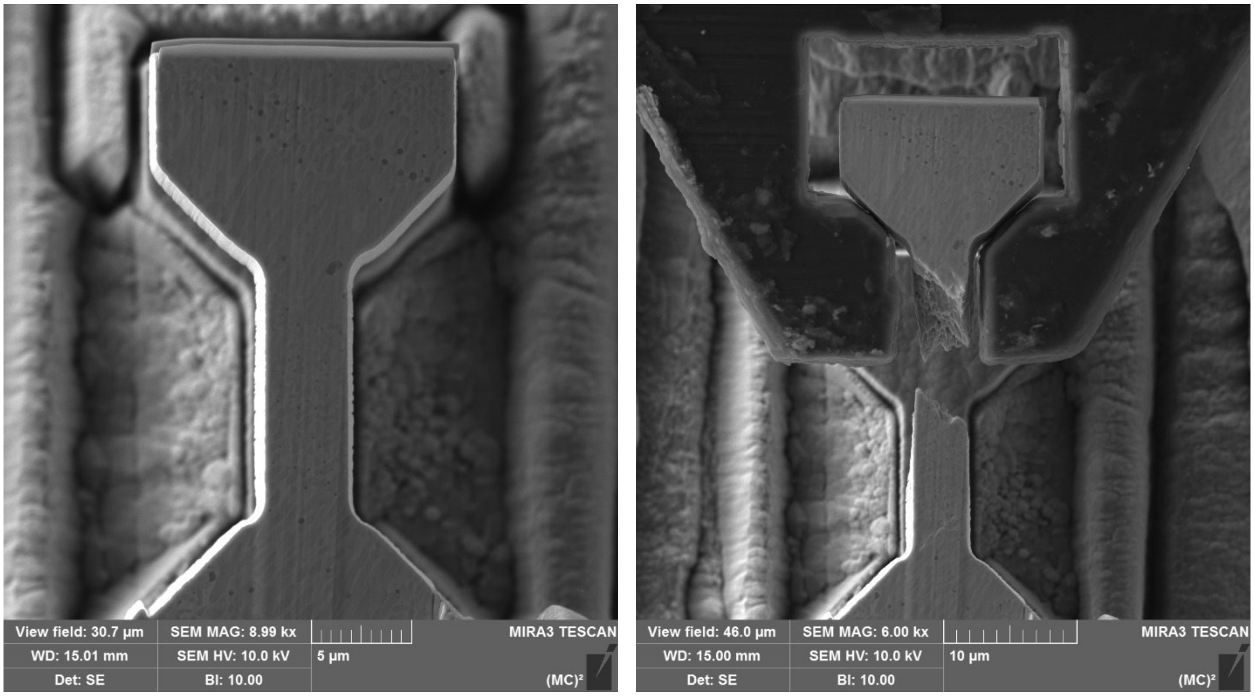

On the left is a SEM image of the finished dogbone before tensile testing. On the right is another image of the dogbone post-fracture.

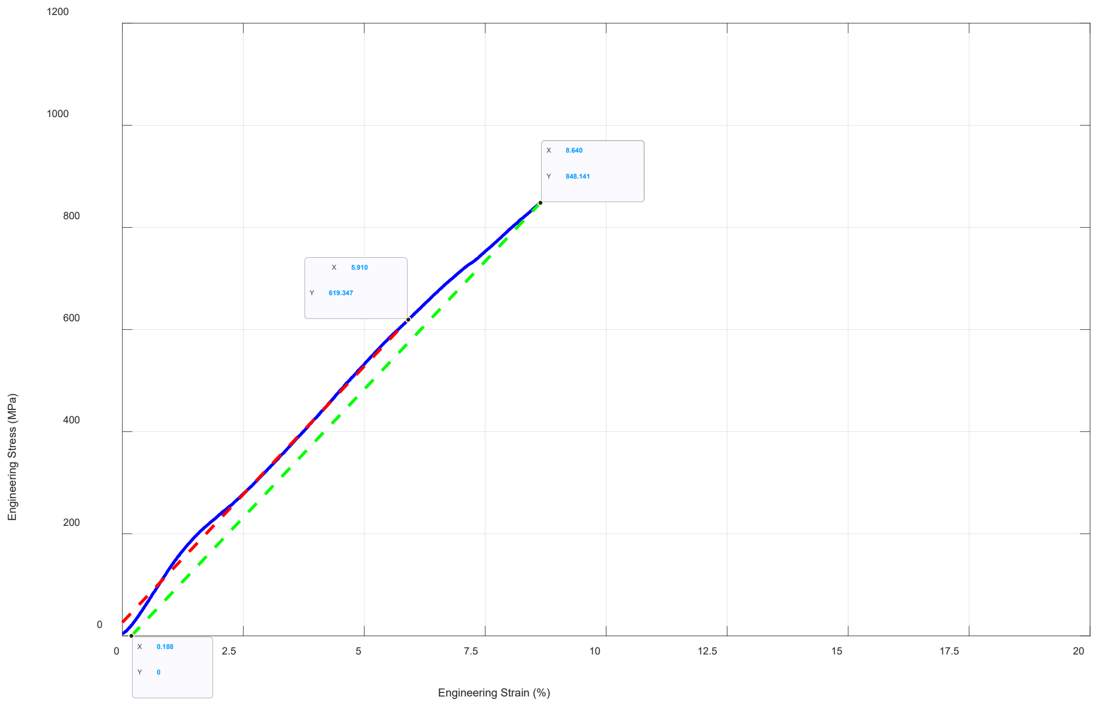

The plot of the engineering stress vs. strain. The plastic strain is identified with an offset curve (dashed green) set at the fracture point that matches the slope of the elastic portion (dashed red).
